# Distribution of Voltage‐Gated Sodium Channels and Scaffolding Proteins on Vestibular Calyx Ending Delineates the Axon Initial Segment

**DOI:** 10.1002/cne.70127

**Published:** 2026-01-28

**Authors:** Anna Lysakowski, Aravind Chenrayan Govindaraju, Steven D. Price, Sophie Gaboyard‐Niay, Irina Calin‐Jageman, Robstein L. Chidavaenzi, Ruth Anne Eatock, Robert M. Raphael, Jay M. Goldberg

**Affiliations:** ^1^ Department of Anatomy and Cell Biology University of Illinois at Chicago Chicago Illinois USA; ^2^ Department of Bioengineering Rice University Houston Texas USA; ^3^ Department of Biological Sciences Dominican University River Forest Illinois USA; ^4^ Department of Biomedical Foundations Ross University, School of Medicine Bridgetown St. Michael Barbados; ^5^ Department of Neurobiology University of Chicago Chicago Illinois USA; ^6^ Department of Pharmacological and Physiological Sciences University of Chicago Chicago Illinois USA

**Keywords:** ankyrin, Caspr, HCN, inner ear, Na channels, RRID:AB_2238842, RRID:AB_2184030, RRID:AB_2040007, RRID:AB_2040009, RRID:AB_177503, RRID:AB_2040202, RRID:AB_2184197, RRID:AB_2184355, RRID:AB_2183861, RRID:AB_2040200, RRID:AB_261544, RRID:AB_477552, RRID:AB_2040204, RRID:AB_2341071, RRID:AB_2301402, RRID:AB_10673165, RRID:AB_2296313, RRID:AB_2115181, RRID:AB_2279449, RRID:AB_10673154, RRID:AB_2083496, RRID:AB_2245198, RRID:AB_10673094, RRID:AB_10673095, RRID:AB_10673030, RRID:AB_10675130, RRID:AB_87580, RRID:AB_2256033, RRID:AB_2271840, RRID:AB_10120130, RRID:AB_90764, RRID:AB_2068506, RRID:AB_94259

## Abstract

The amniote inner ear contains an unusual type of hair cell and a unique postsynaptic calyx terminal with specialized ion channel expression and afferent transmission mechanisms. The calyceal afferent terminal enwraps the hair cell and leads to a heminode. It has morphological and functional microdomains with distinct complements of potassium channels and scaffolding proteins. Stimulation of hair cells gives rise to postsynaptic potentials in the membrane facing the hair cell that propagate along the outer face of the calyx and parent axon to the heminode, giving rise to spikes with timing and response properties that vary with location (epithelial zone) and afferent morphology (calyx‐only vs. dimorphic with additional bouton terminals). Heminodes of calyx‐only afferents lie within the epithelium, placing the calyces themselves closer to the heminode. We report that diverse voltage‐gated sodium (Na_V_) channel proteins (including Na_V_1.1–1.3, 1.5. 1.6, 1.8, and 1.9), HCN (hyperpolarization‐activated cyclic nucleotide‐gated) channels, and associated scaffolding proteins (ankyrins, βIV‐spectrin, and ezrin) are differentially deployed across calyx microdomains, and specific complements of proteins also vary with innervation zone in vestibular epithelia. Our results suggest the calyx outer surface plays a role analogous to an axon initial segment in central neurons, and that systematic variation in Na_V_ pore‐forming subunits underlies differences in firing properties of vestibular afferents in different epithelial zones.

## Graphical abstract

Various sodium channel isoforms and their scaffolding proteins are found in four different microdomains in vestibular calyx‐bearing afferents. These microdomains serve different purposes, analogous to the heminode, axon initial segment, and synaptic domains of other types of sensory afferents.

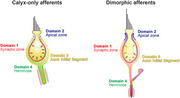

## 1 Introduction

**FIGURE 1:**
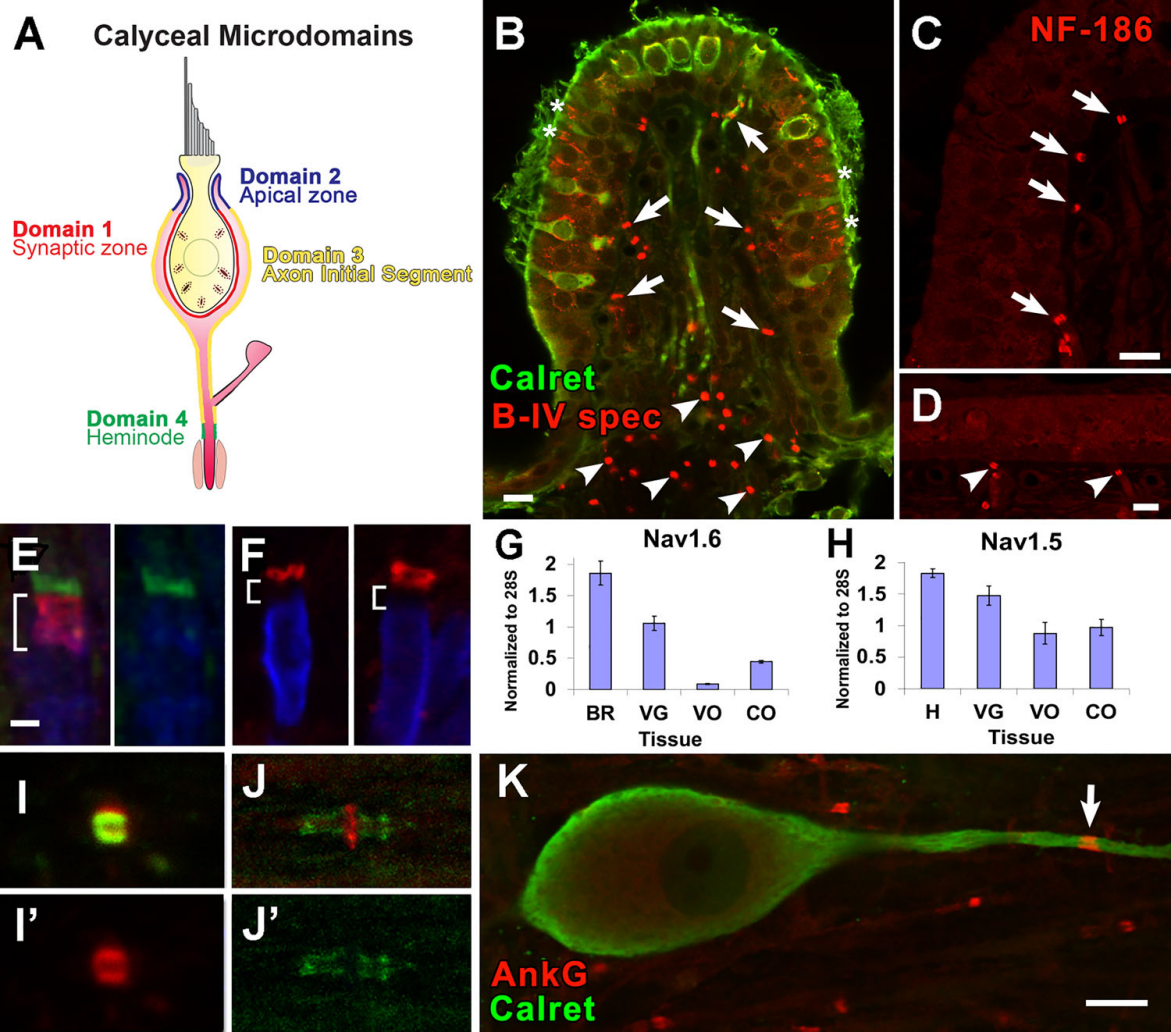
Nodal protein‐like immunoreactivity reveals heminodes and first nodes on the distal process of vestibular afferents. (A) Schematic of calyceal microdomains (modified from Lysakowski et al. 2011). (B) β‐IV spectrin (*B‐IV Spec*, *red*) labels heminodes (Domain 4) and nodes in vestibular afferents and the upper portion of the calyx (Domain 2, *asterisks*) in dimorphic afferents (indicated by a lack of calretinin (*Calret*) immunolabel, *green*). (C,D) Neurofascin‐186 (*NF‐186*, red) labels heminodes (*arrows*) and nodes in crista (*C*) and otolith (*D*) organs. (E) Na_V_1.6 (*green*), the most common nodal Na channel isoform, is present at the heminode, while Caspr (*red*) labels the hemi‐paranode (*brackets*). In the right half of this panel, myelin basic protein (*MBP*, *blue*) extends over the hemi‐paranode to the heminode. (F) Two different examples of ezrin labeling (*red*). Ezrin labels the heminode, MBP (*blue*) labels the internode, and there is a small gap between them as the myelin attenuates, the hemi‐paranode (*brackets*, not immunolabeled here, but seen in panel (*E*), labeled with Caspr, *red*). (G, H) qPCR demonstrates the presence of Na_V_1.6 (*G*) and Na_V_1.5 (*H*) in vestibular ganglion (*VG*) cells (*G, H*), but to a lesser extent in the vestibular (*VO*) and cochlear (*CO*) sensory organs. Brain (*Br*) and heart (*H*) served as controls for Na_V_1.6 and Na_V_1.5, respectively. Values are means ± SEM. (I, I’) Ezrin (*red*) and AnkG (*green*) are seen to be co‐extensive in a rat vestibular nerve node, while ezrin and AnkB (J, J’) are complementary, with ezrin (*red*) labeling the node and AnkB (*green*) the paranode. (K) Vestibular ganglion cell labeled with calretinin (*green*) and Ank G (*red*) labeling the first proximal node of Ranvier (*arrow*). Scale bars: in B‐D, K = 10 µm; in E (also applies to F, I, J) = 2 µm.

Vestibular afferents are bipolar neurons, with the cell body (soma) generating central and peripheral processes. The peripheral branches have characteristics of both dendrites (receiving synapses) and axons (generating and propagating action potentials [APs]). The peripheral terminals can take the form of large calyces (Figure 1A) or boutons. Afferents may be calyx‐only (*C*), dimorphic (*D*, having both types of terminals), or bouton‐only. In this article, we focus on the calyx‐bearing afferents. Turtle afferents, which comprise a greater proportion of bouton‐type afferents, may have different distributions of Na channel isoforms (Holmes et al. 2017), but most markers that we used in this article (see Table 1) did not label small branches or boutons, with the one exception being ankyrin B (AnkB), a scaffolding protein. Vestibular afferent terminals synapse with hair cells that transduce head movement and transmit motion signals to the neuron. The calyces mediate both quantal transmission (Holt et al. 2007; Songer and Eatock 2013; Sadeghi et al. 2014) and an unusual non‐quantal form of transmission (NQT) via currents through presynaptic (hair cell) and postsynaptic (calyx inner face—CIF) ion channels (Holt et al. 2007; Lim et al. 2011; Contini et al. 2017, 2012, 2020; Songer and Eatock 2013; Highstein et al. 2014; Spaiardi et al. 2020; Govindaraju et al. 2023) and generate graded potentials that subsequently initiate APs. Understanding AP initiation in the vestibular calyx requires knowledge of the ion channel composition in the different regions of this unusual synapse in order to account for biophysical processes in the respective compartments that are exposed to different extracellular environments (Govindaraju and Raphael 2025).

**TABLE 1:** Antibody provenances.

			Controls					
Antibodies/antisera	Source	Catalog/RRID no.	Tested in KO?	Immuno	IP	Western	Pre‐absorp	References
Na_V_ Channels								
Na_V_1.1 (ms)	NeuroMab	75‐023 RRID:AB_2238842		X (Br)		X		Datasheet
Na_V_ 1.2 (ms)	NeuroMab	75‐024 RRID:AB_2184030		X (Br)		X		Datasheet
Na_V_1.3 (rb)	Alomone	ASC‐004 RRID:AB_2040007		X (DRG)		X	X (w)	Datasheet
Na_V_1.4	Alomone	ASC‐020 RRID:AB_2040009				X		Datasheet
Na_V_ 1.5 (rb)	Chemicon/Millipore	AB5493 RRID:AB_177503		X (V, H)		X	X (w)	Datasheet; Wooltorton et al. (2007)
Na_V_ 1.6 (rb)	Alomone	ASC‐009 RRID:AB_2040202		X (SN, Br, DRG)		X	X (w, if)	Black et al. (2002)
Na_V_ 1.6 (rb)/(ms)	Rasband/NeuroMab	gift, 75‐026 RRID:AB_2184197	X (if)	X (ON, Br)		X		Rasband et al. (2003); datasheet
Na_V_1.7	NeuroMab	75‐103 RRID:AB_2184355				X		Datasheet
Na_V_1.8	NeuroMab	75‐166 RRID:AB_2183861				X		Datasheet
Na_V_1.9	Alomone	ASC‐017 RRID:AB_2040200						
Pan Na_V_ (rb)	Sigma	S6936 RRID:AB_261544				X		Datasheet
Pan Na_V_ (ms)	Sigma	S8809 RRID:AB_477552		X (ON, SN, Br)		X		Datasheet; Rasband et al. (1999)
Pan Na_v_ (rb)	Alomone	ASC‐003 RRID:AB_2040204		X (Br)		X	X (w)	Datasheet
Pan Na_V_ (ms)	Levinson	Gift						Rasband et al. (1999)
Na_V_ beta subunits								
Na_V_ beta 1	Alomone	ASC‐041 RRID:AB_2341071				X		Datasheet
Na_V_ beta 4	NeuroMab	75‐198 RRID:AB_2301402				X		Datasheet
K_V_ Channel subunits								
K_V_ 1.1 (ms)	NeuroMab	75‐007 RRID:AB_10673165	X (w)	X (Br)		X		Datasheet
K_V_ 1.2 (ms)	NeuroMab	75‐008 RRID:AB_2296313	X (w)	X (Br)		X		Datasheet
KCNQ2‐N1 (rb)*urea	Cooper	gift		X (Br)	X	X	X (w, if)	Cooper et al. (2001); Devaux et al. (2004)
KCNQ3‐C1 (rb)	Cooper	gift		X (Br)	X	X	X (w)	Cooper et al. (2000)
KCNQ4 (rb)	Jentsch	gift	X (w)	X		X		Kharkovets et al. (2000, 2006)
KCNQ5 (rb)	Jentsch	gift		X (cells)		X		Tzingounis et al. (2010)
**HCN channels**								
HCN1	NeuroMab	75‐110 RRID:AB_2115181						Datasheet
HCN2	NeuroMab	75‐111 RRID:AB_2279449						Datasheet
Structural Proteins								
Contactin (ms)	NeuroMab	75‐038 RRID:AB_10673154		X (ON)		X		Datasheet
Caspr1 (ms)	Rasband/NeuroMab	gift, 75‐001 RRID:AB_2083496	X (w)	X (Br)		X		Rasband and Trimmer (2001); datasheet
Caspr2 (ms)	NeuroMab	75‐075 RRID:AB_2245198	X (w)	X (Br)		X		Datasheet
Caspr2 (rb)	US Biologicals	C2089‐70				X		Datasheet
Caspr3 (rb)	Peles	gift		X (Br)		X		Spiegel et al. (2002)
Caspr4 (rb)	Peles	gift		X (Br)		X		Spiegel et al. (2002)
AnkyrinB (rb)/(ms)	Bennett/NeuroMab	gift, 75–144; 75–145 RRID:AB_10673094, RRID:AB_10673095	X (w, if)	X (Br)		X		Scotland et al. (1998); datasheet
AnkyrinG (rb)/(ms)	Bennett/NeuroMab	gift, 75–146; 75–147 RRID:AB_10673030, RRID:AB_10675130		X (Br, ON)				Kordeli et al. (1995); datasheet
βIV Spectrin (rb)	Rasband	gift	X (if)	X (Br, PNS)		X	X	Lacas‐Gervais et al. (2004)
Pan‐Neurofascin	Rasband	gift		X (ON)		X	X	Schafer et al. (2004)
Neurofascin 186 (ms)	Gow/Rasband	gift		X (SN)		X		Schafer et al. (2006)
Ezrin (ms)	Zymed/Invitrogen	35‐7300 RRID:AB_87580		X (cells)				Datasheet
Tenascin‐C (rb)	Chemicon/Millipore	AB19013 RRID:AB_2256033		X (H, V)			X	Huss et al. (2010); datasheet
Tenascin (gt)	Santa Cruz	sc 9871 RRID:AB_2271840						Datasheet
Neurofilament 165 (ms)	DSHB	2H3		X (SC)				Dodd et al. (1988)
**Myelin Basic Protein**								
MBP (ms)	Covance	SMI‐99P RRID:AB_10120130		X (Br)		X		Datasheet
**Calretinin**								
Calretinin (gt)	Chemicon/Millipore	AB1550 RRID:AB_90764		X (Br, Rt)		X	X (w)	Desai, Zeh, and Lysakowski (2005); datasheet
Calretinin (rb)	Chemicon/Millipore	AB5054 RRID:AB_2068506		X (Br)		X	X (w)	Datasheet
Calretinin (ms)	Chemicon/Millipore	MAB1568 RRID:AB_94259		X (Br)		X		Datasheet

Abbreviations: Br, Brain; DRG, dorsal root ganglia; gp, guinea pig; gt, goat; H, Heart; if, immunofluorescence; Immuno, immunohistochemistry; ip, immunoperoxidase; IP, immunoprecipitation; KO, knockout; ms, mouse; ON, Optic Nerve; PNS, peripheral nervous system; Pre‐absorp, preabsorption; rb, rabbit; Rt, Retina; SC, spinal cord; SN, Sciatic Nerve; V, Vestibular Tissue; w, Western; Western, Western blot.

In other systems, such as retinal ganglion or auditory brainstem (E. J. Kim et al. 2019; Raghuram et al. 2019), it is possible to identify the region where synaptic inputs are converted to spike discharge by the distribution of ion channels and scaffolding proteins. Typically, this is at the axon hillock or the unmyelinated axon initial segment (AIS) within tens of microns in distance from the soma. The impact of electrotonic properties and sodium channel densities has been investigated in central neurons (Baranauskas et al. 2013; Gulledge and Bravo 2016; Lazarov et al. 2018). In cortical neurons, the impact of AIS location on AP shape and bandwidth of the dynamic gain function in electrotonically (morphologically) realistic neurons with hypothetical ion channel complements has also been modeled (Verbist et al. 2020). The above approaches require experimental knowledge of where Na^+^ influx occurs to construct biophysical models in order to understand the contribution of ion channels and cable properties in AP initiation (Baranauskas et al. 2013).

In contrast to central neurons, vestibular ganglion afferents are myelinated throughout their length—including the soma itself—except for their central and peripheral terminals. Thus, they do not have a typical AIS adjacent to the soma. Moreover, the shape of the peripheral terminals raised the concern that following hair bundle stimulation, excitatory postsynaptic potentials (EPSPs), triggered by glutamate release from the hair cell (Sadeghi et al. 2014) or NQT could be attenuated (Goldberg 1996) as they disperse toward the heminode where myelination starts (Goldberg 2000; Lysakowski et al. 2011). Given these concerns, the calyx terminal was a candidate location, akin to an AIS, for voltage‐gated sodium (Na_V_) channels that help initiate and shape APs. However, knowledge of Na_V_ channel distribution in calyx membranes is limited, and whether isoform expression varied with afferent morphology (*C* vs. *D*) and zone (central [CZ] vs. peripheral [PZ]) in vestibular sensory epithelia is unclear.

Our earlier article (Lysakowski et al. 2011) delineated the expression of K_V_ channels in different microdomains of the calyx, but our coverage of Na_V_ channels was brief. Here, we examine Na_V_ channel distribution with a pan‐Na_V_ antibody, antibodies to Na_V_ 1.1–1.9 α‐subunit isoforms, and Na_V_β4. We also investigate the expression of AnkB and AnkG, scaffolding proteins that hold Na_V_ and K_V_ channels in the calyx plasmalemma, and HCN channels, which are involved in regulating K^+^ and Na^+^ concentration at calyx synapses.

Our work shows that Na_V_ channels in vestibular calyces are found along the CIF, calyx outer face (COF), and the heminode; that Na_V_ isoform expression varies between calyx‐only and dimorphic afferents; and that these isoforms are akin to those at the AIS. We also found that myelin sheaths that penetrate the neuroepithelium mostly belong to calyx‐only (*C*) fibers that are calretinin‐positive (Desmadryl and Dechesne 1992; Desai, Zeh, and Lysakowski 2005; Desai, Ali, and Lysakowski 2005). Our overall results are consistent with the emerging view that Na_V_ channels in vestibular afferents are more diverse than previously supposed (Liu et al. 2016) and support persistent, resurgent (Meredith and Rennie 2020; Baeza‐Loya and Eatock 2024), and transient (Kalluri et al. 2010; Liu et al. 2016) currents. Further characterization of currents, by isoform, will allow refinement of models of spiking in calyx terminals and should help explain the dynamic diversity of vestibular afferents (Paulin and Hoffman 2019).

## 2 Materials and Methods

### 2.1 Animals

125 Harlan Long‐Evans adult rats of both sexes (100 F, 11 M, 14 not noted), weighing 200–500 g, were obtained from Charles River (Wilmington, MA) and used in these experiments. No differences in immunostaining were noted between the sexes. All animal procedures were approved by the Institutional Animal Care and Use Committee (IACUC) of the University of Illinois at Chicago.

### 2.2 Choice of Antigens

We studied the distribution of *Na_V_* channels in calyx endings, in the unmyelinated axon, and in the heminode of vestibular‐nerve afferents. We used a pan‐*Na_V_* antibody, one β‐subunit antibody, and antibodies to isoform‐specific Na_V_ α‐subunits, including Na_V_1.5, the latter of which had been shown to be present in rat vestibular calyces in the first postnatal month (Wooltorton et al. 2007) and in immature VGN somata (Liu et al. 2016); Na_V_1.6, localized in the AISs of auditory afferents (Hossain et al. 2005; K. X. Kim and Rutherford 2016) and in most central neurons (see, e.g., Katz et al. 2018); Na_V_ 1.2, found at AISs of many central nervous system (CNS) neurons (Boiko et al. 2003; Osorio et al. 2005; Hu et al. 2009; Tian et al. 2014); Na_V_ 1.1, found in the AIS of inhibitory GABAergic CNS neurons (Ogiwara et al. 2007; Duflocq et al. 2008; Lorincz and Nusser 2008) and in auditory nerve fibers (K. X. Kim and Rutherford 2016) and Na_V_1.8, found in vestibular ganglion cells (Liu et al. 2016). Because not all calyx domains labeled by pan‐Na_V_ antibodies were positive for these four antibodies, we also used *Na_V_* antibodies that had not previously been described in hair‐cell afferents: Na_V_1.3, Na_V_1.4, Na_V_1.7, and Na_V_1.9. mRNA expression of Na_V_1.1 ‐ 1.9 had been detected in rat vestibular ganglion at P1 and all except Na_V_1.4 were also found at P21 (Wooltorton et al. 2007). Some of these subunits (Na_V_1.5‐1.8) have also been found by mRNA analysis in human fetal vestibular tissue (Quinn et al. 2021).

We used markers commonly found at nodes of Ranvier, including βIV‐spectrin (Figure 1B) and neurofascin (Figure 1C,D). Myelin basic protein (MBP, Figure 1E,F) also helped to define the heminodes. For scaffolding proteins, we chose antibodies of proteins known to tether *Na_V_* and *K_V_* channels at or near nodes of Ranvier (Hedstrom et al. 2007; Ogawa and Rasband 2008; Rasband and Peles 2021): ezrin (Figure 1F,I,J), AnkB (Figure 1J) and AnkG (Figure 1I,K). We investigated the cell adhesion molecules Caspr1 (Figure 1F) and Caspr2 (see below), which are located in the paranodes and juxtaparanodes, respectively, in other neurons (Lai and Jan 2006; Rasband and Peles 2021). Finally, we chose to look at some candidate hyperpolarization‐activated cyclic nucleotide‐gated channels (HCN1 and HCN2) that carry a mixed K^+^ and Na^+^ current and were thought to be involved in non‐quantal transmission (Meredith et al. 2012, 2023; Horwitz et al. 2014; Sadeghi et al. 2014; Contini et al. 2020, 2017; Govindaraju et al. 2023).

### 2.3 Antibodies

For details on antibody provenances, see Table 1. Antibodies were obtained from several vendors, including Chemicon (Temecula, CA), Alomone (Jerusalem, Israel), Sigma, Inc. (St. Louis, MO), US Biological (Swampscott, MA), Covance, Inc. (Princeton, NJ), and the UC Davis/NIH NeuroMab Facility (Davis, CA). We also obtained antibodies as gifts from Matthew Rasband and Edward Cooper of the Baylor College of Medicine, Thomas Jentsch of the Max‐Delbrück‐Centrum for Molecular Medicine, S. Rock Levinson of the University of Colorado at Denver, and Vann Bennett of Duke University. The rabbit polyclonal antiserum against pan‐Na_V_ was made to an epitope common to all α‐subunits of voltage‐gated Na^+^ channels. Secondary antibodies were either made in donkey against rabbit, goat and mouse IgGs (obtained from Chemicon, AP182R, AP180F, and AP192S, respectively), or we used similar Alexa dye‐labeled secondary antibodies obtained from Molecular Probes (Eugene, OR). Goat anti‐mouse IgG isotype‐specific Alexa dye‐labeled secondary antibodies were obtained from Invitrogen (Carlsbad, CA).

### 2.4 Immunohistochemistry and Confocal Microscopy

Adult Long‐Evans rats of both sexes were deeply anesthetized with sodium pentobarbital (80 mg/kg), then perfused transcardially with 100 mL of physiological saline containing heparin (1000 IU), followed by 2 mL/g body weight of fixative (4% paraformaldehyde, 1% acrolein, 1% picric acid, and 5% sucrose in 0.1 M phosphate buffer [PB] at pH 7.4). Vestibular epithelia were micro‐dissected in PB. In some experiments with Na_V_ antibodies, rats were perfused with only 4% paraformaldehyde and 5% sucrose in 0.1 M PB or were fixed by intralabyrinthine injection of 10% methanol in MES buffer (2‐[N‐Morpholino]ethanesulfonic acid hydrate, “Good's buffer”; Sigma, Cat No. PHG0003‐100G). Otoconia were dissolved with Cal‐Ex (Fisher Scientific, Pittsburgh, PA) for 10 min. Background fluorescence was reduced by incubating organs and ganglia in a 1% aqueous solution of sodium borohydride for 10 min, and tissue was cryo‐protected in 30% sucrose‐PB overnight at 4°C. Frozen sections (35 µm) were cut on a sliding microtome.

Immunocytochemistry was performed on free‐floating sections. Tissues were first permeabilized with 4% Triton X‐100 in phosphate‐buffered saline (PBS) for 1 h at room temperature (RT), then incubated with 0.5% Triton X‐100 in a blocking solution of 0.5% teleost fish gelatin (Fisher Scientific) and 1% bovine serum albumin (BSA) in PBS for 1 h at RT. Ethanol‐ or methanol‐fixed tissues had no permeabilization pre‐treatment, although 0.5% Triton X‐100 was sometimes included in the antibody diluent. Retrieval of methanol‐fixed tissue was improved by post‐fixing the sections in 4% paraformaldehyde after antibody incubations. Sections were incubated with a cocktail of two, or in some cases three, primary antibodies for 72 h at 4°C. Most primary antibodies were diluted to 1:200 in the blocking solution, except for Caspr1 (1:250), βIV‐spectrin (1:400), AnkB (1:2000), and the Sigma pan‐Na_V_ (1:100). We used calretinin antibody as a marker of type II hair cells and of *C* afferents (Desmadryl and Dechesne 1992; Desai, Zeh, and Lysakowski 2005; Desai, Ali, and Lysakowski 2005).

Specific labeling was typically revealed by incubating sections in a cocktail of two secondary antibodies (fluorescein‐conjugated donkey anti‐goat IgG, and rhodamine‐conjugated donkey anti‐rabbit IgG, diluted 1:200 in blocking solution) for 24 h at 4°C. If a third primary antibody was used, typically Cy5‐conjugated donkey anti‐mouse IgG was used with fluorescein or Alexa 488‐conjugated donkey anti‐goat IgG, and rhodamine‐ or Alexa 594‐conjugated donkey anti‐rabbit IgG. To combine two different mouse monoclonal antibodies (all NeuroMab antibodies are mouse monoclonals) in the same experiment, we used IgG isotype‐specific secondary antibodies (Invitrogen, Carlsbad, CA). Sections were rinsed with PBS between and after incubations, and mounted on slides in Mowiol (Calbiochem, Darmstadt, Germany).

### 2.5 Immunogold Electron Microscopy (EM)

We investigated the ultrastructural localization of some markers with immunogold EM, and we present results here for Na_V_1.9 and AnkG. Vestibular epithelia were sectioned at 40 µm with a Vibratome 2000 (Technical Products International). Free‐floating sections were permeabilized with 0.5% Triton X‐100 for 1 h, then blocked in a solution consisting of 0.5% fish gelatin and 1% BSA for 1 h. Sections were incubated in primary antibody (1:50 dilution) for 72 h, rinsed, then incubated for 24 h in secondary antibody (1:40 dilution), tagged with ultra‐small (0.8 nm) colloidal gold‐labeled F(ab) goat anti‐mouse IgG and goat anti‐rabbit IgG (Aurion, Cat. No. 25413, EM Sciences, Hatfield, PA). Colloidal gold staining was silver‐enhanced (4–8 min; Silver IntenSE M kit, Cat. No. RPN491; GE Healthcare, Waukeska, WI). Sections were dehydrated in a graded series of alcohols and propylene oxide, embedded in Araldite (Fluka Durcupan, Cat. No. 14040, EM Sciences, Hatfield, PA) on glass slides with plastic coverslips, and polymerized at 55°C for 48 h. The section of interest was cut free from the slide, glued on top of a blank Araldite block, ultrathin‐sectioned at 70 nm with a diamond knife (DiATOME, Quakertown, PA), and stained with uranyl acetate and lead citrate. Sections were examined and photographed with a JEOL 1220× transmission electron microscope.

### 2.6 Antigen Retrieval Methods

Because sodium channel antibodies are notoriously difficult to work with in aldehyde‐fixed tissues, we used several different antigen retrieval methods, including microwave, pepsin or trypsin digestion, urea incubation, boiling tissue in citrate buffer, as well as ethanol and methanol fixation.

### 2.7 Controls

In addition to the various controls listed in Table 1, we performed “no primary antibody” controls with each experiment, which revealed a lack of non‐specific staining by the primary and secondary antibodies. Positive control tissues, like spinal cord and optic nerve, were reacted alongside vestibular tissue and used for nodal proteins. We also confirmed expression of some of the Na_V_ subunits by performing standard RT‐PCR (data not shown) or quantitative RT‐PCR (qPCR) for Na_V_1.5 and Na_V_1.6 on whole vestibular ganglia and end organs (brain or heart tissue used as controls). Briefly, RNA from adult rat tissue was isolated, reverse transcribed, and qPCR was performed using primers for Na_V_1.6 (Du et al. 2007) and Na_V_1.5 (Shang et al. 2008). Amplifications were performed in triplicate, and the 28S rRNA gene expression was used for normalization. In previous studies from our labs (Wooltorton et al. 2007; Liu et al. 2016), all Na_V_ α‐ and β‐subunits, except Na_V_1.4, have been demonstrated with RT‐PCR to be present in vestibular ganglia in P21 rats.

### 2.8 Image Analysis

Slides were examined on a laser‐scanning confocal microscope (LSM 510 META, Carl Zeiss, Oberköchen, Germany) and images were obtained with Zeiss's ZEN software. Final image processing and labeling was done with Adobe Photoshop (San Jose, CA). Some whole macular organs were processed for various combinations of antibodies; Z stacks of optical sections were then obtained on the confocal microscope, with the thickness of the optical sections varying from 0.4 to 1.0 µm. Some Z‐stacks were deconvolved and reconstructed in 3D with VOLOCITY software (v. 3.7, Improvision, Lexington, MA). In some instances (noted in the figure legends), using the ZEN software, a maximum intensity projection image was produced from a Z‐stack of images. To determine exact locations of the basement membrane for heminodal location analyses, we temporarily adjusted the brightness of an image to visualize it and drew a dashed line overlying its location.

## 3 Results

To determine the overall distribution of Na_V_ alpha subunits in calyx‐only (*C*) and dimorphic (*D*) fibers, we used pan‐Na_V_ antibodies. Na_V_ channels were broadly distributed on CIFs, COFs, and heminodes. Subsequently, specific antibodies were used to study the distribution of individual subunits (Na_V_1–Na_V_9). We then investigated the presence of ankyrins and HCN. The ankyrins (AnkG and AnkB) are scaffolding proteins that fix the position of Na_V_ channels and other molecules in the plasmalemma of axons (V. Bennett and Lorenzo 2013; Yoshimura and Rasband 2014). HCN channels affect non‐quantal transmission by regulating the concentration of both Na^+^ and K^+^ ions in the synaptic cleft, which sets the resting potential of both the hair cell and calyx (Meredith et al. 2012, 2023; Biel et al. 2009; Horwitz et al. 2014, 2011; Contini et al. 2017; Govindaraju et al. 2023).

### 3.1 Definition of Calyx Microdomains

In describing calyx endings, we use the terms *distal* and *proximal*, respectively, for locations further from or closer to the brainstem. These structures were divided into the same domains (see Figure 1A) defined by (Lysakowski et al. 2011). *Domain 1* lies in the bottom of the CIF, directly opposite the many ribbon synapses in the enclosed type I hair cell. *Domain 2* lies on both sides of the distal tip of the calyx ending in *D* units. In *C* units, which have shorter calyx terminals, this domain may be truncated or absent. *Domain 3* consists of the rest of the COF (*3a*) and unmyelinated sections of the parent axon or stalk (*3b*) extending toward *Domain 4*, the heminode, which is located just distal to the start of myelination.

### 3.2 Distribution of Pan‐Na_V_ Immunostaining

**FIGURE 2:**
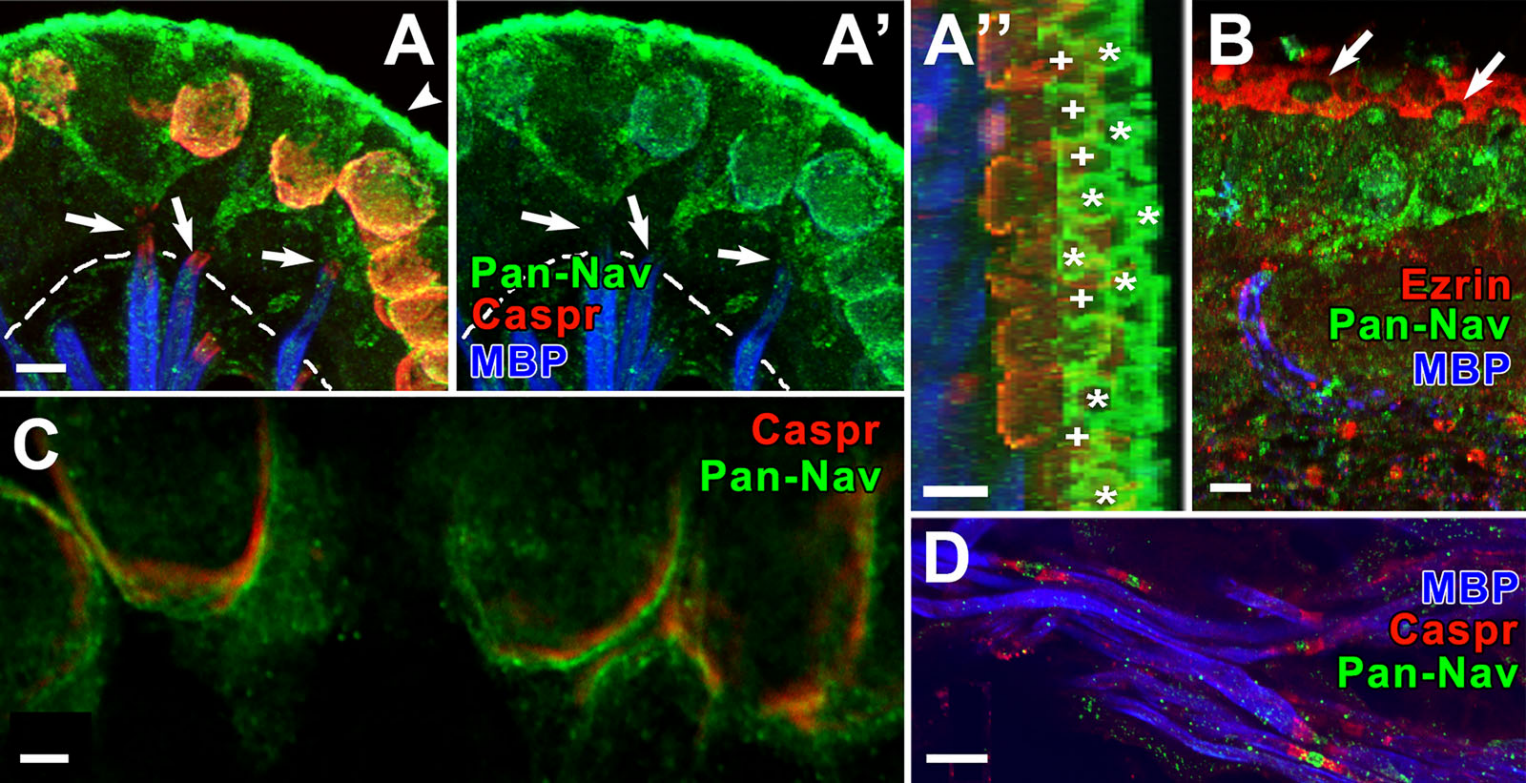
Pan‐Na_V_ antibody labels all four domains within calyx endings in rat vestibular organs. (A, A’) In this maximum intensity projection image, several labeled calyces and their parent axons are visualized with a Pan‐Na_V_ antibody (*green*, same color conventions apply to all A panels). Caspr1 (*Caspr*, *red*) labels Domain 1 and the para‐heminode (*arrows*) on each afferent fiber. *MBP* (*blue*) labels the myelin of the afferent fibers, which gets progressively thinner as it ends over the para‐heminode (see Figure 1E). Dashed lines indicate the basement membrane (BM), visualized by temporarily increasing the brightness of the image. *A’* is the same image as *A*, with the Caspr1 (*red*) label removed, illustrating presence of pan‐Na_V_ (*green*) on calyx endings and parent axons. (A″) Top of the same stack as in *A* and *A′* (*arrowhead in A indicates point of view for image stack rotation*), showing that the apical layer of the neuroepithelium is composed of circular structures (asterisks,*), presumably the tops of calyx endings (Domain 2); adjacent structures outline more rectilinear spaces (*plus signs*, *+*), marking the tops of supporting cells. (B) Different stack from a utricular macula illustrates pan‐Na_V_ label (*green*) in several calyces. Ezrin (*red*) labels the microvilli on supporting cells, in addition to the nodes and heminodes of afferents in the stroma (*arrowheads*). MBP (*blue*) labels stromal afferent fiber myelin. Because the stack is tilted, the tops of four calyces (Domain 2, *arrows*) can be seen through holes in the ezrin‐labeled microvilli of the supporting‐cell layer. (C) Single section from crista CZ shows pan‐Na_V_ (*green*) immunostaining on calyx outside the Caspr1 label (*red*). (D) Pan‐Na_V_ (*green*) in nodes and Caspr1 (*red*) in paranodes of several myelinated fibers labeled with MBP (*blue*) in the stroma of a saccular macula. Scale bars: in A, A”, B, D = 5 µm; in C = 2 µm.

A pan‐*Na_V_* antibody was used in combination with a Caspr1 antibody. Caspr1 (Figure 2A) has previously been used to mark the CIF (Domain 1, Sousa et al. 2009; Lysakowski et al. 2011); it is a cell adhesion protein that anchors K channels in membranes, particularly at paranodes (Salzer 2003), the part of the axon immediately adjacent to nodes of Ranvier. Na_V_ labeling labeled the CIF (Domain 1) of each calyx terminal (Figure 2A’), continued onto the COF (Domains 2 and 3), and extended to the heminode (Domain 4). Overlap of red (Caspr1) and green (pan‐Na_V_) produced orange labeling on the CIF (Figure 2A). Intense pan‐Na_V_ labeling at the apical surface of the neuroepithelium in sectioned material (Figure 2A,A’) suggested non‐specific labeling because sometimes antibodies exhibit a so‐called “edge effect.” However, rotating the stacks to show three‐quarter top views (Figure 2A, B) revealed a circular pattern of Na_V_ staining consistent with its localization in the tops of calyx endings, not the entire apical surface. We later observed a similar pattern of apical calyx labeling for the Na_V_1.9 isoform (see below). Pan‐Na_V_ labeling outside the Caspr1 labeling, including the COF, was commonly seen in single sections (Figure 2C). Pan‐Na_V_ antibodies, as expected, also labeled nodes of Ranvier (Figure 2D) in the stroma, which were flanked by paranodes immunostained for Caspr1.

### 3.3 Differences in Heminodal Location of *C* and *D* Units

**FIGURE 3:**
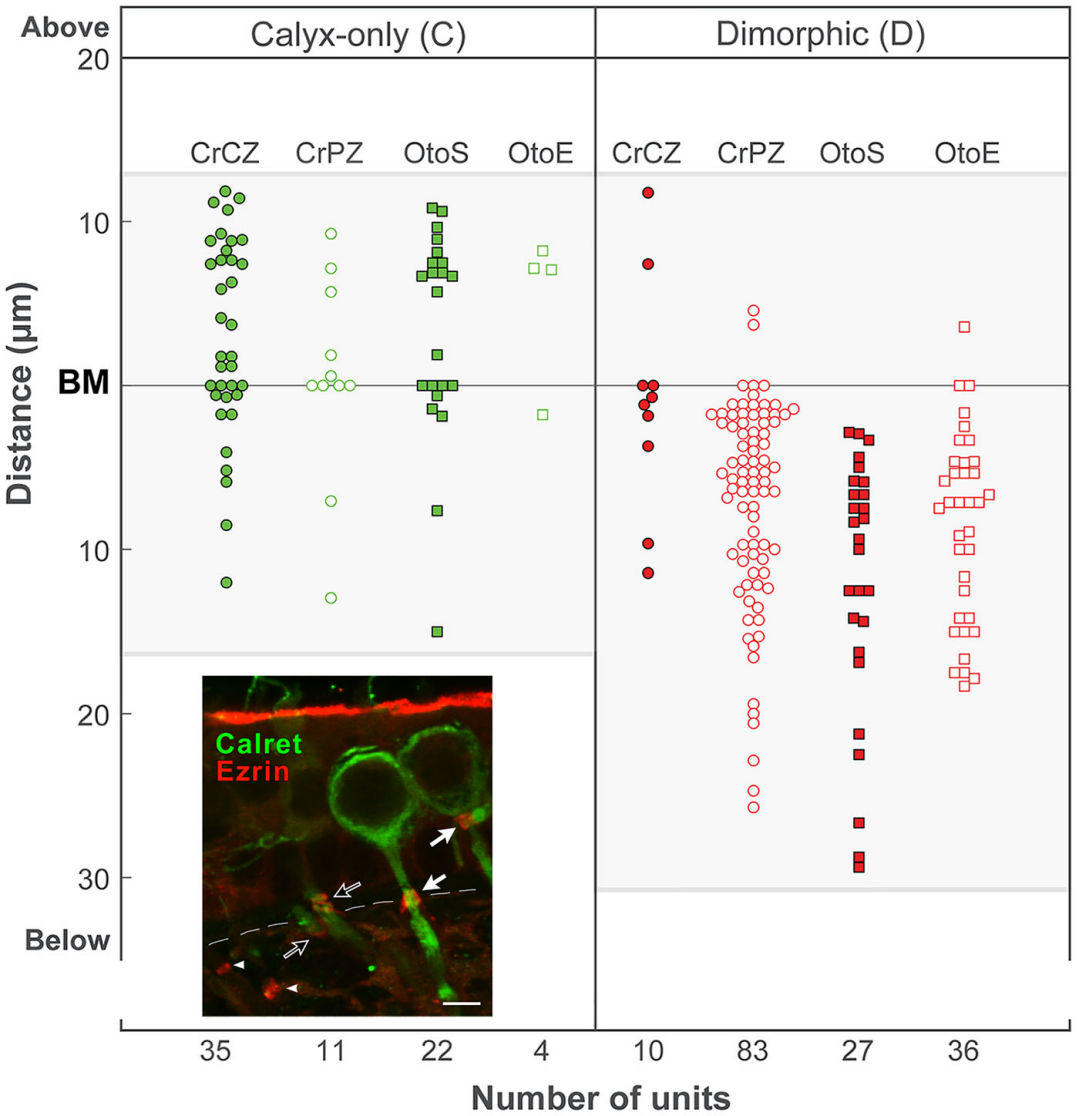
Heminodes of calyx‐only afferents in the central/striolar zones are closer to the calyx. Heminodal locations relative to the basement membrane (see *Inset*) for calyx‐only (*C*, *green*) and dimorphic *(D*, *red)* fibers in the crista ampullaris central and peripheral zones (*CrCZ* and *CrPZ*, respectively) and otolith striolar and extrastriolar zones (*OtoS* and *OtoE*, respectively); *abscissa*, distances above and below the basement membrane (*BM*). *C* fibers were distinguished from *D* fibers with calretinin (*green*) immunohistochemistry. Data are based on 228 (72 *C* and 156 *D*) fibers. Heminodes were identified with MBP combined with heminodal markers such as ezrin, KCNQ3, βIV‐spectrin, or AnkB, the latter a marker of the hemi‐paranode. *Inset, lower left*, Two calyces in an adult female utricular macula, from *C* fibers *(closed arrows)* labeled with calretinin (*green*) and marked with ezrin (*red*), which overlie the heminodes located above the basement membrane (*dashed line*). Ezrin also labels the microvilli on the apical surfaces of supporting cells. The calyces for the two calretinin‐labeled (*green*) fibers in the center of the figure (*open arrows*) are largely out of the plane of section and their ezrin‐labeled (*red*) heminodes are seen just above or below the basement membrane. Two other ezrin‐labeled heminodes *(arrowheads)* are seen in the stroma at the left lower corner; these heminodes presumably belong to dimorphic fibers as they are not labeled with calretinin. Scale bar = 5 µm.

A survey was done of the locations of heminodes and myelin in 228 *C* and *D* afferents (Figure 3). Calretinin immunostaining, specific for *C* rather than *D* fibers (Desmadryl and Dechesne 1992; Desai, Zeh, and Lysakowski 2005; Desai, Ali, and Lysakowski 2005), was used as the *C* afferent marker. As *C* fibers are centrally located, calretinin is also a marker for the crista central (CrCZ) and otolith striolar (OtoS) zones of vestibular epithelia. Figure shows ezrin labeling of several afferents in a utricular macula; ezrin typically marks the end of myelination at each fiber's heminode (Chang and Rasband 2013). Heminodes of *C* fibers were usually located within the neuroepithelium, whereas heminodes of *D* fibers were usually found below the basement membrane in the stroma. In Figure 3 (*inset*), on the right side, two *C* afferent calyx endings have heminodes located above the basement membrane, and the rightmost of the two heminodes abuts its calyx ending. The two calretinin‐positive *C* fibers in the middle of the inset have their calyx endings out of the section plane; their heminodes are located just above or below the basement membrane. Two ezrin‐positive heminodes, seen to the left, are located below the basement membrane; the lack of calretinin immunostaining indicates that they belong to dimorphic afferents.

**FIGURE 4:**
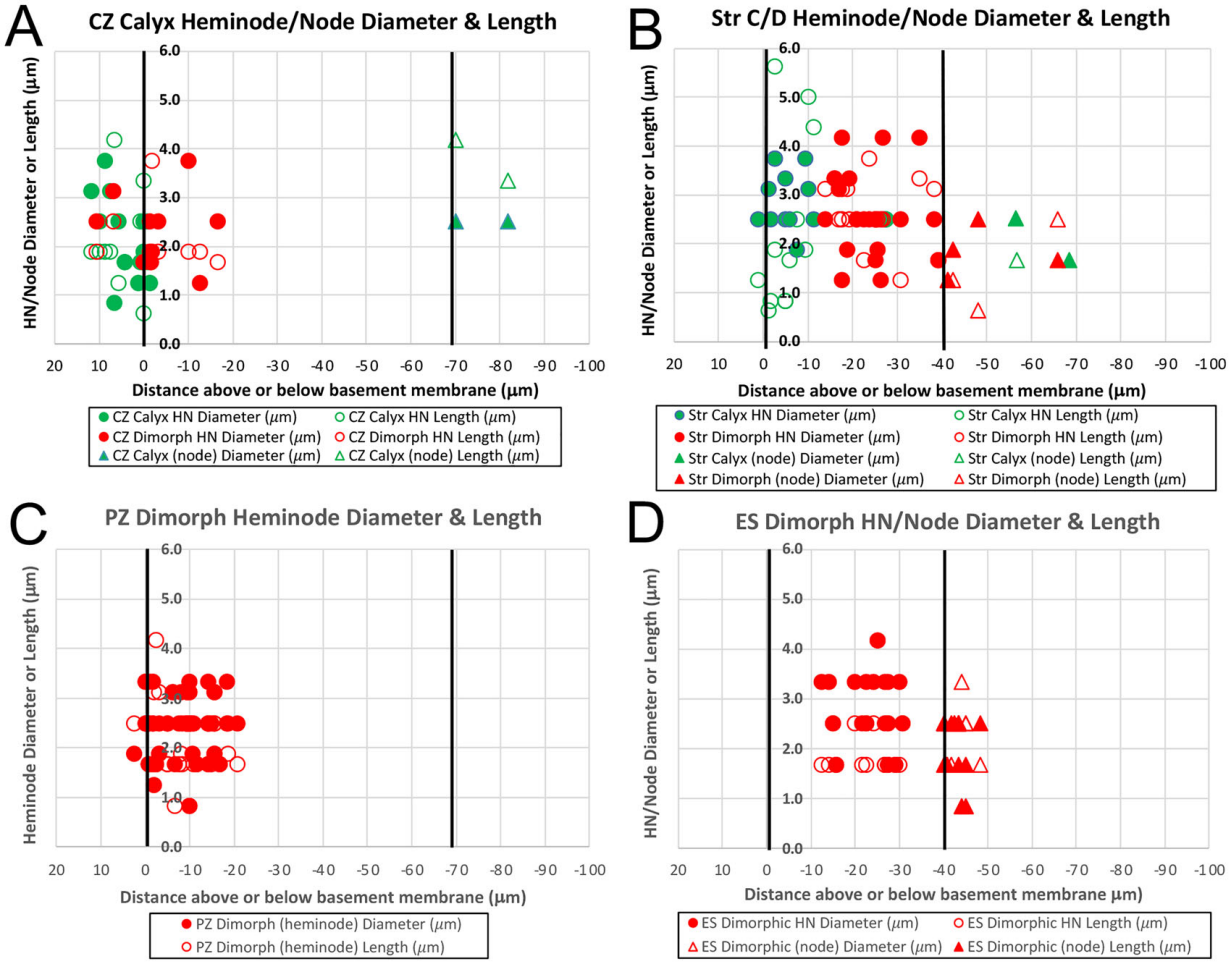
Distributions of heminodes (HNs) and nodes of calyx‐only and dimorphic afferents in crista and otolith organs. (A) Distance from the basement membrane (*BM*) in crista central zone (*CZ*) calyx‐only (*C*, *green*) and dimorphic (*D*, *red*) afferents versus heminodal (HN) and nodal diameter and length. The bottom of the calyx generally sits at about 15 µm above the basement membrane. *Left vertical line* marks the basement membrane. *Right vertical line* marks the cut‐off distance between HNs and nodes. Conventions hold for all 4 panels. Data are based on 140 (31 *C* and 109 *D*) fibers. There was a significant difference between *C* and *D* units in distance above the BM (Student's *t*‐test, *p* < 0.05). (B) Otolith striolar zone (*Str*) calyx‐only (*C*) and dimorphic (*D*) afferents versus heminodal diameter and length. The difference in distance below BM between these two afferent classes was very highly significant (Student's *t*‐test, *p* <<< 0.00001). (C) Crista peripheral zone (*PZ*) dimorphic (*D*) afferents versus HN diameter and length. (D) Otolith extrastriolar zone (*ES*) dimorphic (*D*) afferents distance from the basement membrane versus HN and nodal diameter and length. For other tests of significance, see text.

In the cristae (Figure 3), the heminodes of *C* afferents were mostly (54/72 = 75%) located in the neuroepithelium. In contrast, only 12/156 = 7.7% of *D* fibers had their heminodes at or above the basement membrane. The neuroepithelial heminodes (54 + 12 = 66) were found at the apex (central zone) of the crista or in the striolar region of each otolith organ, precisely where *C* afferents are found (Fernández et al. 1988, Fernández et al. 1990; Desai, Zeh, and Lysakowski 2005; Desai, Ali, and Lysakowski 2005). *D* fibers are found throughout the cristae or maculae. A 4 × 2 contingency test with Yates correction, comparing fiber type (*C* vs. *D*), zonal location (striolar or CZ vs. extrastriolar or PZ), and heminodal location (above vs. below the basement membrane) indicates that the results are highly significant (χ^2^ (3df, *N* = 228) = 95.0825, *p* <<< 0.001). Student's *t*‐tests found highly significant differences between striolar *C* versus *D* in terms of heminodal distance relative to the basement membrane, with *C* fiber heminodes on average 6.8 µm below the basement membrane (BM) versus striola *D* at 24.8 µm below (*N* = 13C, 20D striolar otolith organ fibers, *p* < 2.9E‐07). In the crista, CZ *C* fiber heminodes, on average, were 4.5 µm *above* the BM versus CZ *D* fibers, which were on average 1.0 µm *below* the BM (*N* = 53*C*, 16*D* CZ crista fibers, *p* < 0.05). PZ *D* fiber heminodes were 9.5 µm *below* the BM (N = 99*D* PZ vs. 53*C* CZ crista fibers, *p* < 2.4E‐16). While calretinin remains a superior marker to identify *C* versus *D* afferents, the results indicate that heminodal location may also be used, albeit with an uncertainty of ≈10%–25%, given that 8% of CZ *D* heminodes were located above the basement membrane and 25% of CZ *C* and all striolar and extrastriolar D heminodes could be found below it (Figure 4).

For 140 fibers, nodal and heminodal diameters and lengths along the axon (Figure 4) were also analyzed. Mean heminodal lengths were significantly longer in striolar *D* than in extrastriolar *D* (strD vs. extstrD, 2.6 µm vs. 1.4 µm, respectively; *N* = 20 strD, 19 extstrD, Student's *t*‐test, *p* < 0.02). Heminodal lengths for striolar *D* (mean 2.6 µm) versus nodal lengths of striolar *D* (1.4 µm) were significantly longer (*N* = 20 strD heminodes, 4 extstrD nodes, Student's *t*‐test, *p* < 0.05).

### 3.4 Isoform‐Specific Na_V_ Immunostaining

Of the various isoforms (Na_V_1.1–1.9), we did not detect Na_V_1.4 or Na_V_1.7 in the vestibular afferents despite finding Na_V_1.7 in our positive‐control, dorsal root ganglion (DRG) tissue. The distribution of other isoforms is presented below.

#### 3.4.1 Domain 1–Na_V_1.5

**FIGURE 5:**
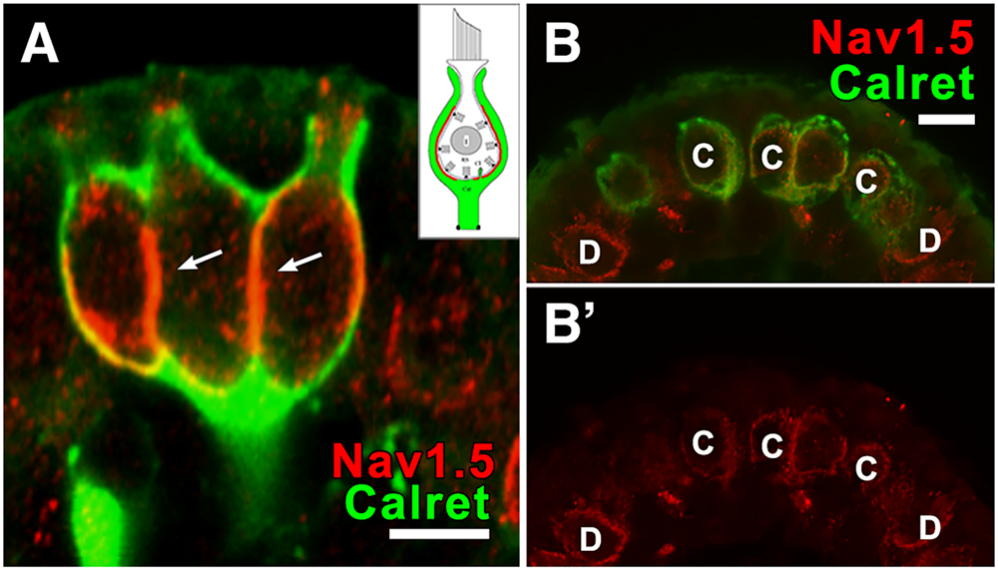
Na_V_ 1.5 marks Domain 1 intensely in young animals, less so in older animals. (A) P14 rat crista: Na_V_1.5 (*red*) in a complex calyx (a calyx enclosing two or more vestibular type I hair cells) afferent ending. Arrows point to internal walls inside this complex calyx. (B, B’) Adult (4‐month‐old female, 300 g) rat crista: Na_V_1.5 *(red)* is present on the inner face of calyx endings in both calretinin‐positive *(green) C* units (*B*) and calretinin‐negative *D* units (*B’*) from the central zone. Scale bars in A, B = 5 µm.

Na_V_1.5 immunolabeling is found on the CIF (Domain 1) of calretinin‐positive *C* endings and calretinin‐negative *D* units (Figure 5). The staining intensity levels observed here in a 4‐month‐old adult female (Figure 5B) are diminished, compared to those found in P14 afferents (Figure 5A). Similar results were seen throughout the neuroepithelium of both cristae and maculae. We saw similarly intense Na_V_1.5 staining levels in P21 rat utricle striolar zone in a previous study (see Figure 12A in Wooltorton et al. 2007).

#### 3.4.2 Domain 2 of *D* Afferents—Na_V_1.9

**FIGURE 6:**
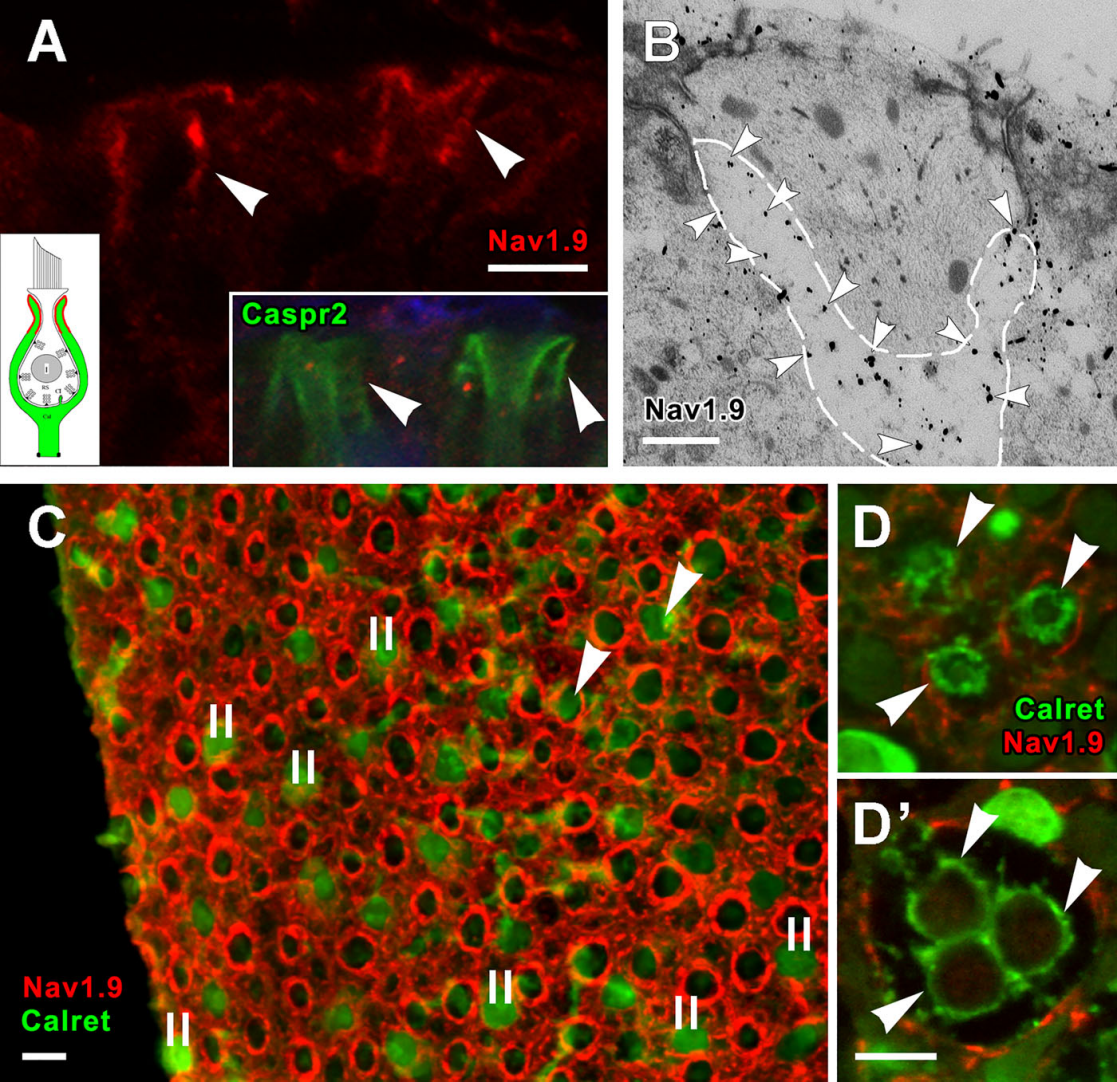
Na_V_1.9 labels Domain 2 in calyx endings of dimorphic afferents. (A) Transverse section of a crista showing two dimorphic calyx endings, whose apical parts (Domain 2) are stained for Na_V_1.9 *(red*, *arrows)*. *Inset, left*, schematic of entire calyx, showing Domain 2 in red. *Inset, right*, immunolabeling of Domain 2 with Caspr2 (*green*, *arrows*) to show similarity to Na_V_1.9 immunolabeling. (B) Electron micrograph showing Na_V_1.9 label (*arrowheads*) in Domain 2, marked by dashed lines, rather than in the enclosed hair cell or the supporting cells. (C) Surface view of the extrastriolar region of a whole mount utricular macula from an adult female rat. Na_V_1.9 antibody (*red circles*) labels the upper portion (Domain 2) of *D* calyx endings. Most type II hair cells (*II*) and a small proportion of type I hair cells are labeled with calretinin (*solid green*, Desai, Zeh, and Lysakowski 2005). (D, D’) A complex calyx, from the striolar region in the same macula as in *A*, is labeled with calretinin (*green*), defining it as a *C* fiber. *D* is more superficial than *D’*. The three calyces in *D* fuse at deeper levels (*D’*) to form one triple complex ending. This and other *C* fibers lacked Na_V_1.9 labeling. Scale bars: *A, C, D* = 5 µm; *B* = 0.2 µm.

Na_V_1.9 labeling is confined to the upper portions (Domain 2) of *D* calyx endings (Figure 6A), similar to Caspr2 immunoreactivity (*right inset*). The localization of Na_V_1.9 is confirmed in EM immunogold sections labeled with the same Na_V_1.9 antibody (Figure 6B); gold particles are concentrated in the upper part of a calyx ending. Na_V_1.9 immunolabeling is present in the upper part of calyx endings of *D* afferents, as can be seen in a surface view of the extrastriolar utricular macula (Figure 6C). Such labeling is absent in the calyx endings of *C* units (Figure 6D,D’), likely because the apical tips of most of their calyx endings are truncated, so as to lack Domain 2.

#### 3.4.3 Domain 3 of *C* Units—Na_V_1.3

**FIGURE 7:**
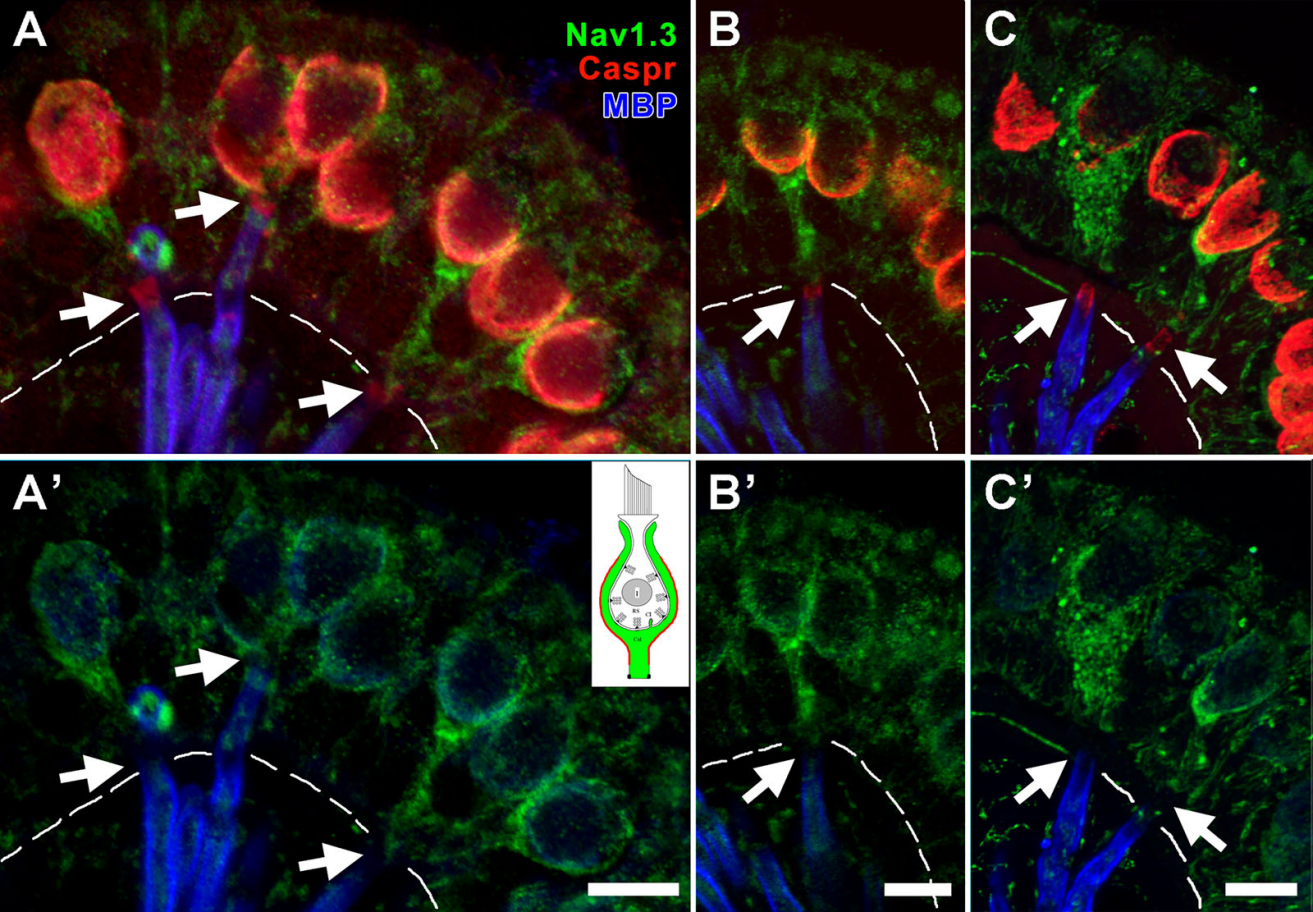
Na_V_1.3 antibody labels Domain 3 in presumed *C* units. (A, A’) In this maximum intensity projection image of a stack from a crista, the presumption is based on the relatively superficial location of Caspr1‐stained hemi‐paranodes *(red, arrows)* and the location of the units in the CZ. Na_V_1.3 labeling (*green* in A’) is absent in the PZ (*not shown*), which contains *D*, but not *C*, units. In the three units marked by arrows in A, MBP (*blue*) can be traced from the parent axons below the basement membrane (BM) *(dashed line) to* the stained hemi‐paranodes. In A’, Caspr1 immunostaining (*red*) has been removed, leaving Na_V_1.3 labeling (*green*) of Domain 3, including the outer surface of the calyx endings (COF, Domain 3a) and the unmyelinated parent axons above the heminodes (Domain 3b, locations indicated by *arrows*). *Inset, upper right*, schematic of entire calyx, showing Domain 3 in red. (B, C) Additional examples of calyx afferents labeled with Na_V_1.3. Same color conventions as in A. Scale bars = 5 µm.

Figure 7A shows the central zone of a crista. Caspr1 immunostains the CIF (Domain 1), and the hemi‐paranodes of the three units in *A (arrows)* and MBP stains their myelin sheaths. The relatively superficial location of these three heminodes suggests that they belong to C fibers. After removal of the Caspr1 channel (Figure 7A’), it is seen that the Na_V_1.3 antibody labels *Domain 3*, the base of each COF, as well as its parent axon. Na_V_1.3 immunostaining is not seen in the crista peripheral zone where calyces all belong to *D* units. This suggests that Na_V_1.3 in *Domain 3* is peculiar to *C* fibers.

#### 3.4.4 Domain 3 of *D* Units and in Nodes of Ranvier—Na_V_1.2

**FIGURE 8:**
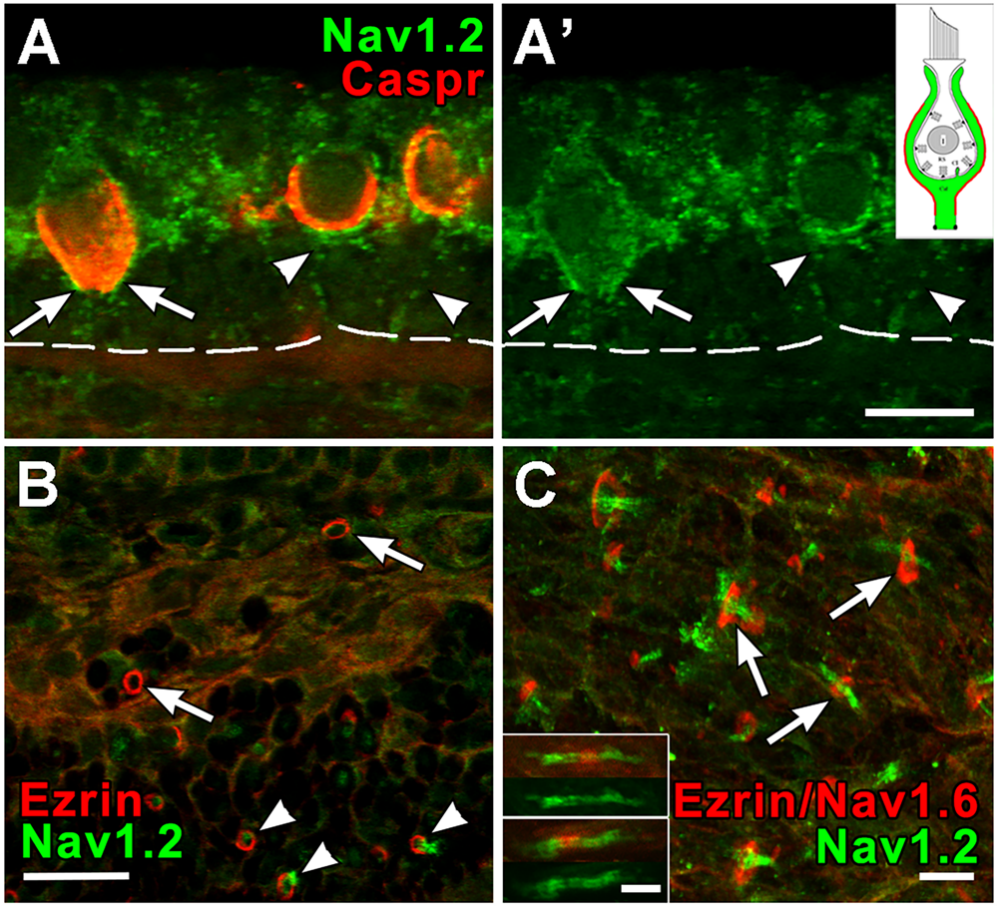
Na_V_1.2 labels Domain 3 in majority of afferents, first nodes, and stromal paranodes. (A, A’) Na_V_1.2 (*green*) labels the calyx outer surfaces (Domain 3, *arrows*) of *D* afferents in the extrastriolar zone of the utricular macula from an adult female rat. The calyx inner surfaces are marked with Caspr1 (*Caspr, red* in A). The beginning of two parent axons *(arrowheads)* can be seen in the neuroepithelium, but could not be traced into the stroma, where the heminodes of most *D* units are located, presumably because they go out of the plane of this single section. *Inset in A’, upper right*, schematic of entire calyx, showing Domain 3 in red. (B) Na_V_1.2 antibody (*green*) labels mostly stromal nodes (*arrowheads*), but not heminodes (*arrows*), both of which are marked with an ezrin antibody (*red*). (C) Deeper in the stroma of the crista in a maximum intensity projection, many nodes can be observed. The Na_V_1.2 (*green*) extends beyond the boundaries of the node, marked by ezrin (*red*). C *insets*. Na_V_1.6 antibody (in this case *red*) immunostains two nodes of Ranvier in the stroma of a rat saccular macula. Similar to (C), Na_V_1.2 (*green*) also stains the nodes (seen after removal of the Na_V_1.6 immunolabel (*red*) in the bottom half of each inset) and extends well beyond each node into the paranode. Scale bars: A–C = 10 µm; C inset = 5 µm.

In a maximum projection image from a utricular extrastriola, the Na_V_1.2 label is present in Domain 3 of D units, including the lower part of the COF and the adjoining (distal) part of the unmyelinated parent axon (Figure 8A,A’). The heminodes do not contain Na_V_1.2, but the first nodes of Ranvier, which are located near the base of the stroma, do contain both Na_V_1.6 and Na_V_1.2 (Figure 8B). The Na_V_1.2 staining extends beyond that of Na_V_1.6 into paranodes. The more extensive Na_V_1.2 staining may be a vestigial feature of the predominance of this isoform in the less compact nodes seen early in axonal development (Boiko et al. 2001; Salzer 2002). More proximal nodes in the vestibular nerve, including those near Scarpa's ganglion, also contain both Na_V_1.6 and Na_V_1.2 (Figure 8C, *inset*).

#### 3.4.5 Domain 4 of *C* and *D* Fibers—Na_V_1.1

**FIGURE 9:**
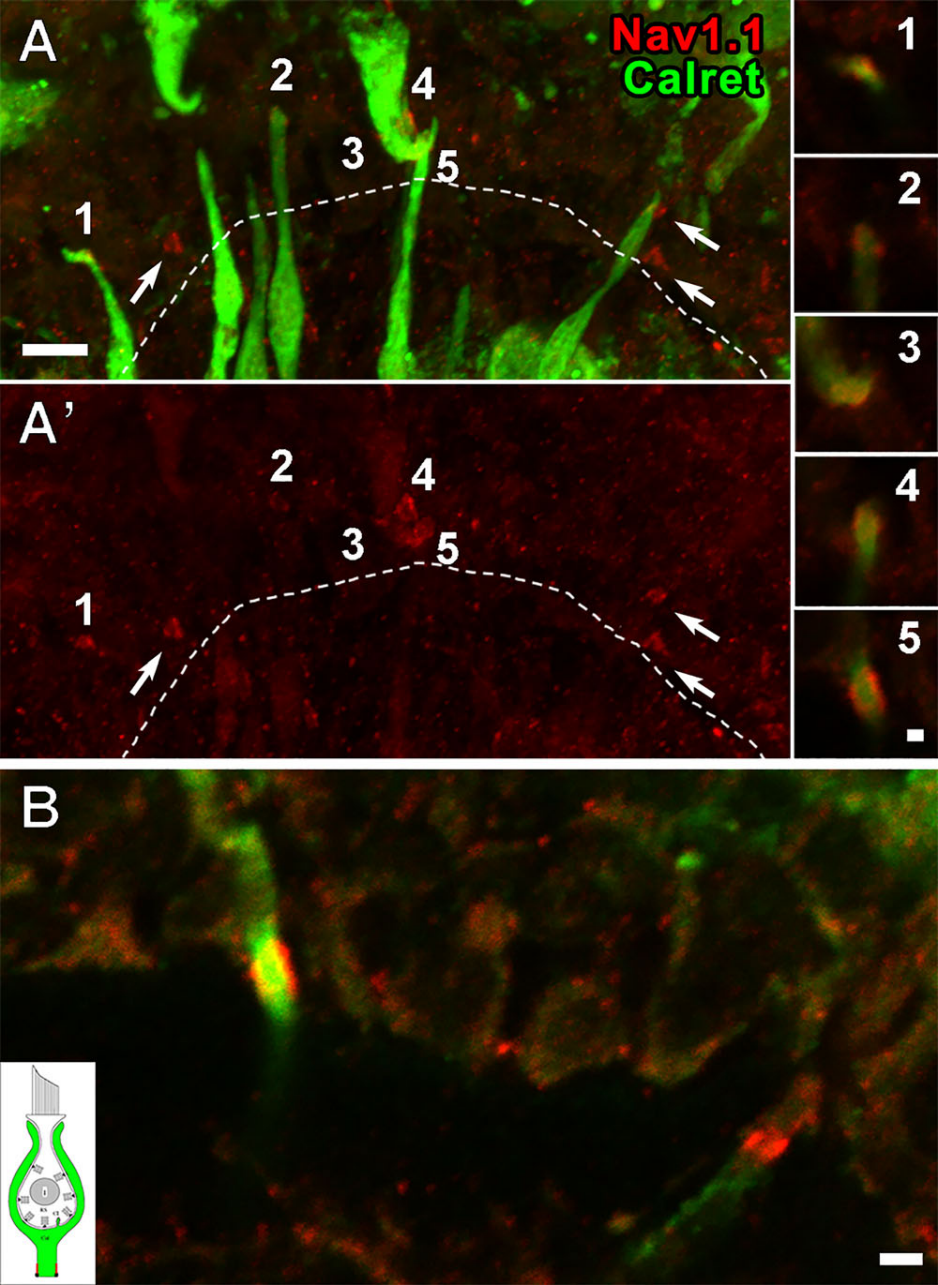
Na_V_1.1 labels heminodes of both calyx‐only and dimorphic afferents. This maximum intensity projection of an image stack was taken from the central zone of an adult female rat crista. The tissue was fixed by intra‐labyrinthine perfusion with a methanol‐based fixative. (A) Calretinin *(green)* stains *C* axons; calyx terminals were not preserved because of the weak fixation. Na_V_1.1 antibody *(red)* stains heminodes in five calretinin‐positive *C *fibers (numbered *1 ‐ 5)* and three calretinin‐negative *D* fibers *(arrows)*. The basement membrane (BM) is indicated (*dashed line*). (A’) The channel containing calretinin labeling has been removed to reveal the Na_V_1.1 labeling. Heminodes of *D* units appear above the BM because the section from which the stack was obtained was cut at a slightly oblique angle. *(Insets, right)*. Higher‐magnification of the five *C*‐fiber heminodes, each stained for Na_V_1.1 *(red)* and calretinin *(green)*; the intensity of the calretinin label has been reduced to visualize the Na_V_1.1 labeling more easily. (B) Higher magnification single image of both the calretinin‐labeled *C* fiber (left) numbered “5” and the upper right non‐calretinin‐labeled *D* fiber (right) in (A, A’) Scale bars: in A = 5 mm (also applies to A’); in A insets = 1 µm; and in B = 2 µm.

In searching for the Na_V_ isoform present in *C* heminodes, we occasionally found Na_V_1.1 immunolabeling there. To enhance Na_V_1.1 immunolabeling, which can be blocked by strong (2%–4%) paraformaldehyde fixation (Duflocq et al. 2008), we replaced our standard 4% paraformaldehyde fixative with a methanol‐based fixative. The latter did not preserve calyx endings (which are very sensitive to anoxia), but did reveal Na_V_1.1‐like immunoreactivity at heminodes of calretinin‐positive *C* afferents and calretinin‐negative *D* afferents (Figure 9). Based on the immunostaining of Figures 4 and 9, *D* heminodes contain both Na_V_1.1 and Na_V_1.6, whereas *C* heminodes contain Na_V_1.1, but not Na_V_1.6.

#### 3.4.6 Domain 4 of *D* Afferents and Nodes of Ranvier—Na_V_1.6

**FIGURE 10:**
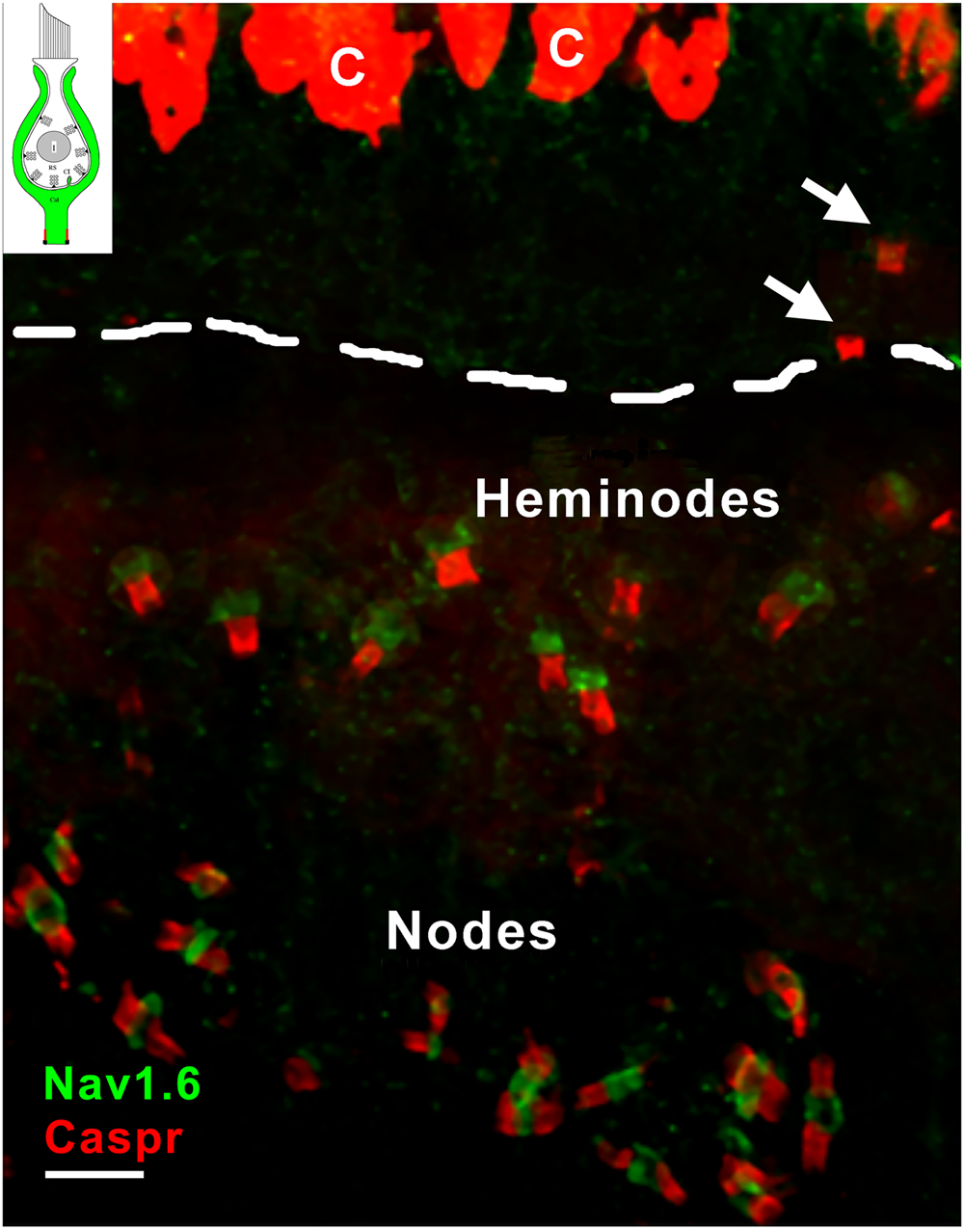
Na_V_1.6 marks both nodes and heminodes, except for heminodes in calyx‐only afferents. Caspr1 (*Caspr, red*) labels the inner surface of calyces, the proximal (central) sides of heminodes (Domain 4, *red* in *schematic, top left*) and in the paranodes on both sides of nodes in this maximum intensity projection image of a utricular macula. Note that all the nodes and most of the heminodes are labeled with Na_V_1.6 (*green*), except for two heminodes (*arrows*), sitting just above the Caspr‐labeled hemi‐paranodes, presumably belonging to C fibers located above the basement membrane (*dashed line*). Scale bar = 5 µm.

Na_V_1.6 is a major isoform involved in spike initiation in other systems (Lorincz and Nusser 2008; Kole and Stuart 2012). Remarkably, Na_V_1.6 is virtually absent in heminodes (Domain 4) within the vestibular sensory epithelium. The two heminodes above the basement membrane (Figure 10), which probably belong to *C* fibers, lacked Na_V_1.6 staining. However, we almost always found Na_V_1.6 in stromal heminodes located below the basement membrane (Figure 10)—heminodes likely belonging to *D* units (cf. Figures 3 and 4). Na_V_1.6 is also present in virtually all nodes of Ranvier, seen near the bottom of Figure 10, as described in other systems (Salzer 2003). Heminodes and nodes were distinguished by differences in Caspr1 staining: heminodes are bordered by Caspr1 only on the proximal side of Na_V_1.6 staining; nodes have Caspr1 staining in the paranodes on both sides of Na_V_1.6. As we never successfully combined calretinin and Na_V_1.6 immunostaining, we are uncertain whether the small percentage (7.7%) of heminodes that do not have Na_V_1.6 and occur above the BM, belong only to *C* fibers. Likewise, we could not ascertain whether Na_V_1.6 is present in *C* fiber heminodes that occur below the BM (25%).

#### 3.4.7 Domain 4 of D Fibers—Na_V_β4

**FIGURE 11:**
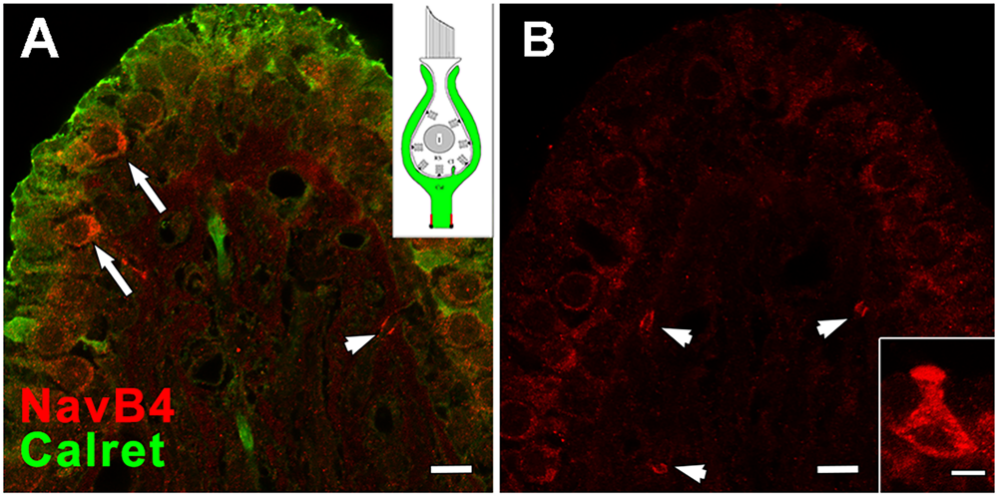
Na_V_β4 accessory subunit found in heminodes of *D* fibers. (A) Section of a rat crista ampullaris, counter‐labeled with calretinin antibody (*Calret, green*) to mark the *C* afferents and type II hair cells. A heminode (*arrowhead*) and three type I hair cells (*arrows*) are observed, labeled with Na_V_β4 antibody (*NavB4*, *red*). (B) Three heminodes (*arrowheads*) marked with an Na_V_β4 antibody (*red*), all located in the stroma and heading into the sensory epithelium in the PZ of an adult crista ampullaris section. *Inset*: From another section, a peripheral type I hair cell is intensely labeled with the same Na_V_β4 antibody. Scale bars: in A, B = 5 µm; in B, inset = 5 µm.

We found Na_V_β4 was present mostly in heminodes in the crista peripheral zone (*N* = 59/76 in PZ, 17/76 in CZ, see Figure 11), but absent from *C* fibers, which were distinguished by calretinin labeling. Thus, most Navβ4 labeling likely occurred on *D* fibers. Some hair cells were also labeled for Na_V_β4; most were type I hair cells, as indicated by their shape and lack of hair cell calretinin labeling.

#### 3.4.8 Domain 4—Na_V_1.8 (Variable)

**FIGURE 12:**
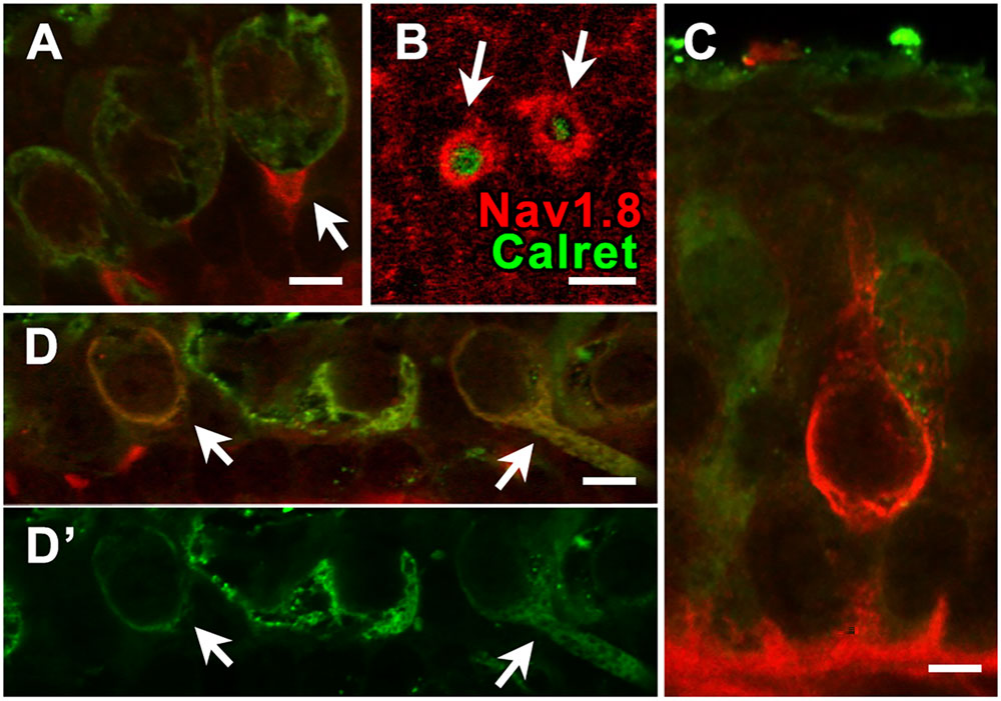
Na_V_1.8 antibody labels vestibular afferents and has three different patterns. (A) The heminodes of calyx‐only afferents (*green*, color conventions are the same in all panels) are densely labeled with Na_V_1.8‐like immunoreactivity (*red*), as seen in both longitudinal section (A) and (B) cross‐section of calyces labeled with calretinin. (C) Occasionally, the inner membrane of a calyx surrounding a type I hair cell, most likely belonging to a dimorphic afferent since these were found in the periphery, is labeled with Na_V_1.8. However, because confocal lacks the resolution of electron microscopy, this could also be a type I hair cell membrane. In (D) Na_V_1.8 is seen to label the calyx endings of calyces (*arrows*) with relatively light calretinin label (D’). Scale bars: A, C, D = 5 µm, B = 10 µm.

An antibody to the Na_V_1.8 isoform labeled the heminode of calyx‐only afferents (Domain 4, Figure 12A,B). In a few instances (see, e.g., Figure 12C), a peripheral type I‐shaped structure, presumably the inner face of a *D* afferent calyx (Domain 1), was labeled. Finally, in some of the lighter calretinin‐labeled calyx endings, Na_V_1.8 labeled the entire calyx (Domain 3, Figure 12D,D’) down to the heminode (out of view).

#### 3.4.9 AnkG in Domains 3 and 4; AnkB in Domain 3 and at Para‐Heminodes

**FIGURE 13:**
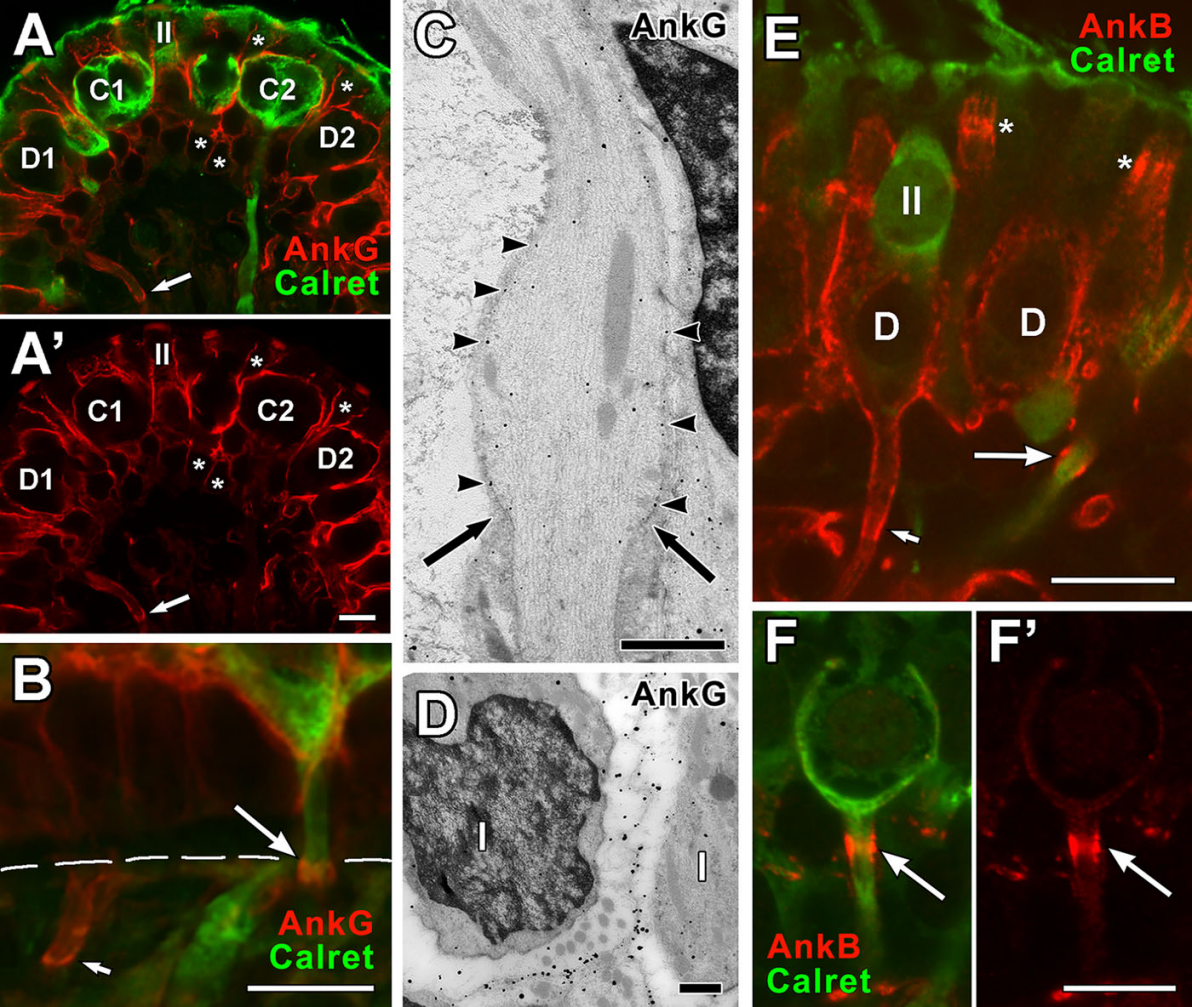
Ankyrin G (AnkG) and Ankyrin (AnkB) are scaffolding proteins for Na and K channels in vestibular epithelia. (A) In a crista, *AnkG* labels calyx endings of calretinin‐positive *C* fibers (*green*, *C1* and *C2*), calretinin‐negative D fibers (*D1* and *D2*) and the lateral membranes of several supporting cells, whose centers are marked by asterisks (*). (A’) Without the calretinin marker, AnkG (*red*) is seen to outline the *C* and *D* calyx endings and both type II hair cells and supporting cells. The parent axon of a calretinin‐negative *D* fiber and its heminode (*long arrow)* are AnkG‐positive. (B) In a utricular macula, AnkG staining labels the heminode (*long arrow*) and the outer face (Domain 3a) of a calretinin‐positive C calyx (*green*); much of the parent axon (Domain 3b) connecting the heminode with the calyx ending lacks AnkG staining. In a *D* fiber on the lower left, the heminode (*short arrow*) and parent axon above it are both AnkG‐positive. (C) Immunogold EM of a crista axon with the termination of the myelin just above the hemi‐paranode indicated (*long arrows*); AnkG label is absent in the hemi‐paranode but is present as gold particles (*arrowheads*) in the heminode. (D) Immunogold EM of two neighboring calyx endings, each enclosing a type I hair cell (*I*); AnkG gold particles are seen on or near the outer surfaces of both endings. (E) Two *AnkB*‐positive *D* calyx endings (*red)* in a crista with one of the endings showing a parent axon and an AnkB‐enhanced hemi‐paranode (*short arrow*). A calretinin‐positive *(green)* calyx‐only fibe*r* with an AnkB‐positive hemi‐paranode *(long arrow)*. Two other enhanced AnkB bands are seen near the tops of two other *D* calyx endings *(asterisks)*, possibly located in Domain 2, where we have also observed βIV‐spectrin (see Figure 1B). (F) A calretinin‐positive *C* calyx (*green*) with an AnkB hemi‐paranode (*red, long arrow*); after removal of the calretinin label (F’), the short parent axon and the calyx ending, unlike the hemi‐paranode, are seen to be barely labeled for AnkB. The AnkB bands marked by arrows in (E), (F), and (F’) are presumably located in paranodes; the location is confirmed in other material, where these bands are located below the heminodes (marked by ezrin) Scale bars: A’ (for A,A’) = 5 µm; in C, D = 0.5 µm; B, E, F, F’ = 10 µm.

We used AnkG and AnkB immunolabeling as proxies for Na_V_ and K_V_ channel localization, respectively. In our material, AnkG was found in calyx endings of both *C* and *D* units; in *D* fibers the labeling was also present along the unmyelinated axonal segments (Domain 3; Figure 13A,A’,B). In both *C* and *D* fibers, AnkG was seen at heminodes (Domain 4) and was confirmed by immunogold EM (Figure 13C); it was also concentrated on the outer faces of calyx endings, likely *D* afferents (Figure 13D). AnkG was relatively absent on the CIF of *D* afferents, despite a very high concentration of K_V_7.4 (KCNQ4) channels in this location (Lysakowski et al. 2011) and despite a known association of K_V_7.2/3 subunits with AnkG (Xu and Cooper 2015). In contrast, in *C* afferents, the bottom part of the COF (Domain 3a) was labeled for AnkG, but the unmyelinated parent axon between the heminode and the calyx ending was not consistently labeled (Domain 3b). Domain 3b in *C* afferents is of variable length, either non‐existent (with the heminode being in direct contact with the calyx ending) or extending as much as 15–25 µm to the heminode situated at, or just below, the basement membrane. AnkG label is also found in the cuticular plate (Figure 13A,A’) and in the region of the striated organelle (Vranceanu et al. 2012), where it likely interacts with β2‐spectrin, a protein component of the striated organelle.

AnkB immunostaining was found at the presumed hemi‐paranode (the K_V_ channel region, immediately adjacent to the heminode, under the myelin, cf. Figure 1I) in *C* afferents. In *D* units, AnkB staining appears to be more intense on the outer calyceal face (Figure 13E) and throughout Domain 3 (3a and 3b) than in *C* afferents COFs (Figure 13F,F’).

#### 3.4.10 HCN Channels in Domains 1–3

**FIGURE 14:**
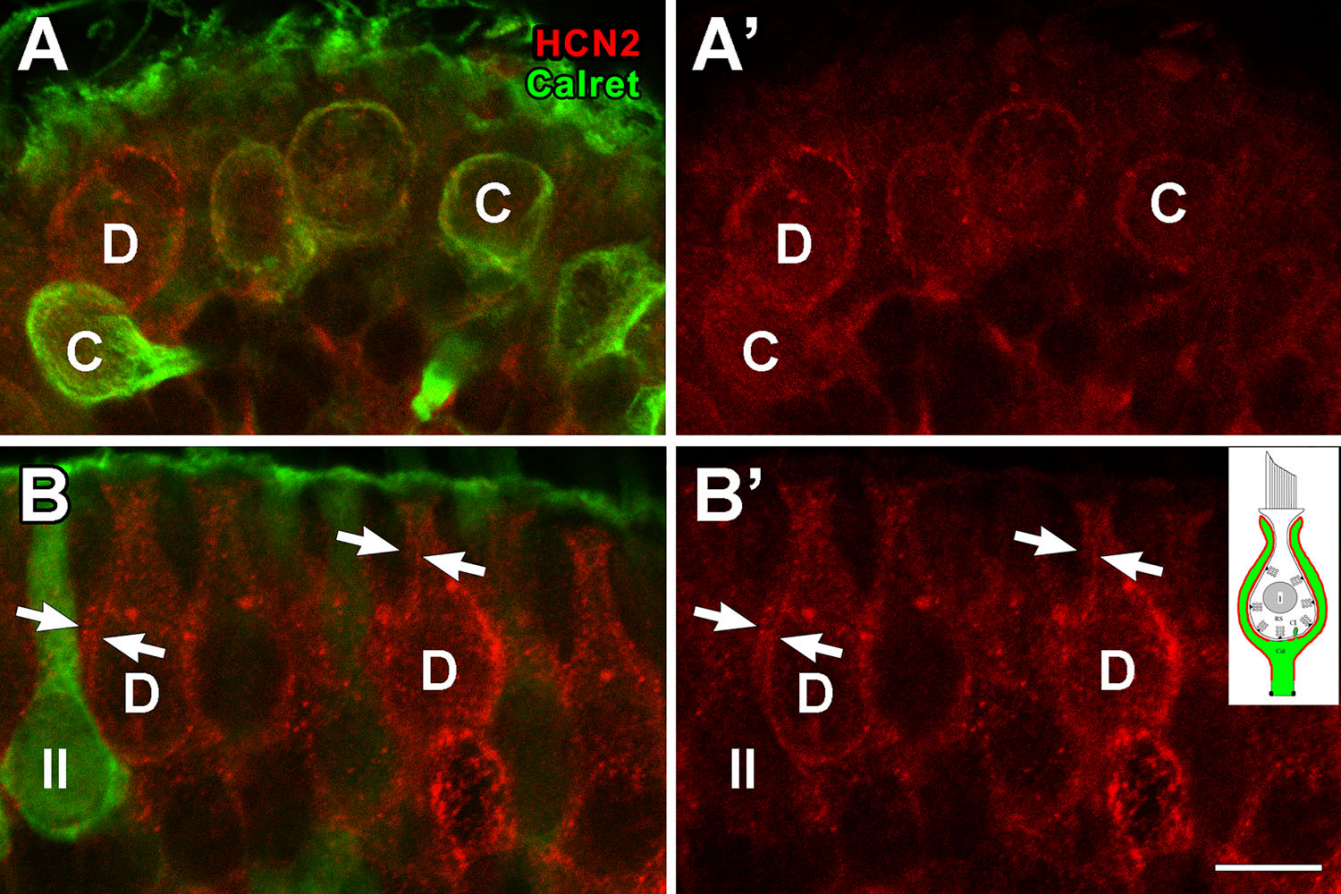
HCN, an I_h_ ion channel, localized to both inner and outer surfaces of calyces. (A) Section from the CZ of a female rat crista immunolabeled for calretinin (*Calret*, *green*) and HCN2 *(red)* in several calretinin‐positive calyx‐only afferents; a single calretinin‐negative dimorphic calyx (*D*) is present. In (A’), the calretinin label seen in (A) has been removed to show light HCN2 labeling of all calyces, including the dimorphic calyx ending. (B, B’) Same immunolabeling as in (A, A’), but from the PZ. A single type II hair cell (*II*) and several dimorphic (*D*) calyces are present; the calyces are slightly more heavily labeled for HCN2 than are those in the CZ; the type II hair cell is very lightly HCN2 labeled. HCN2 label is found on both the inner and outer surfaces of the calyx (*arrows*). Scale bar (for all four panels) = 5 µm.

HCN1 was found in hair cells (data not shown) as previously described (Horwitz et al. 2014). HCN2 is present in all calyx endings on both CIF and COF surfaces; the labeling is slightly heavier in the PZ (Figure 14B,B’), as compared to the CZ (Figure 14A,A’).

## 4 Summary of Results

**FIGURE 15:**
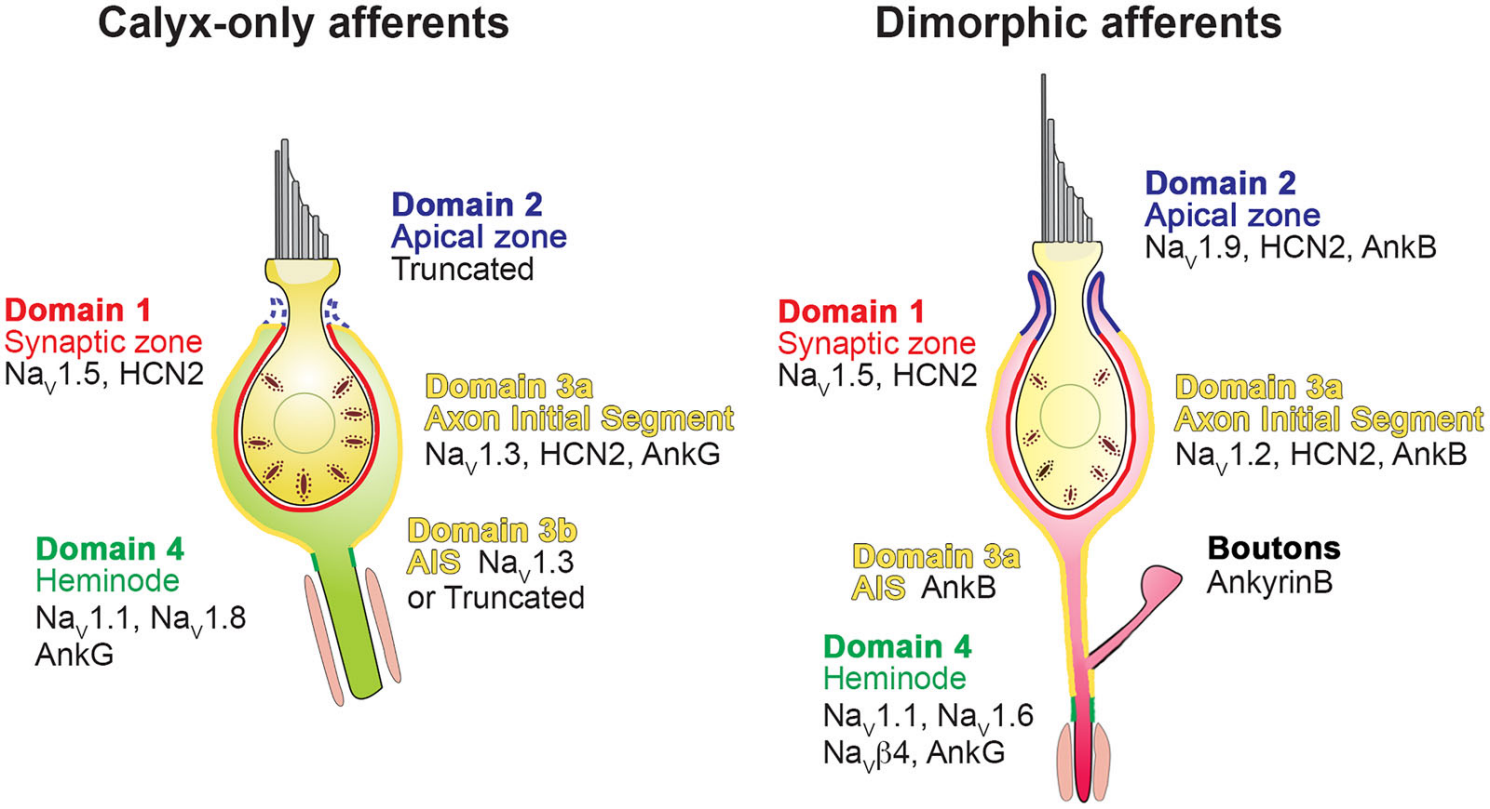
Sodium channel isoform distribution in calyx‐only and dimorphic afferents. *Domain 1* is located on the inner surfaces of both kinds of calyx endings (calyx‐only and dimorphic). *Domain 2*, at the distal tip of the calyx, is present in *D* afferents but truncated in *C* afferents. *Domain 3* consists of the outer face of the calyx ending (*3a*) and the unmyelinated part of the parent axon (*3b*) interposed between the calyx ending and *Domain 4*, the heminode. Note that the boutons and thin collaterals on the *D* afferents did not label with any Na^+^ (or K^+^) ion channel isoforms in our study.

Calyx endings in vestibular cristae and macular organs have several Na_V_ channel isoforms (except for *Na_V_1.4* or *Na_V_1.7*), each localized to a restricted surface domain or domains (Figure 15). *Na_V_1.1* appears at heminodes (*Domain 4*) of both *C* and *D* units; *Na_V_1.2* occupies *Domain* 3 in *D* units, including the unmyelinated axon segment below the calyx ending and the lower part of the COF. Analogously, *Na_V_1.3* is present in *Domain* 3 of *C* units. A *Na_V_1.4* signal was not observed. *Na_V_1.5* is present in *Domain* 1 on the CIF of most units. *Na_V_1.6* occurs only at *D* heminodes and nodes of Ranvier; we also observed *Na_V_β4* at *D* heminodes. *Na_V_1.7* was not seen. *Na_V_1.8* labeling was variable, sometimes at the heminodes (*Domain 4*) and in some cases present along an entire calyx terminal. *Na_V_1.9* is expressed in *Domain* 2 near the tops of calyx endings in *D* units but is lacking in many *C* unit endings. The scaffolding proteins AnkG and AnkB also had differential expression across the surface domains. We found *HCN2* on both CIF and COF surfaces (*Domains 1–3*) of *C* and *D* units. Overall, our results were similar for the cristae and the maculae.

## 5 DISCUSSION

### 5.1 Distinct Domains and Roles for Na_V_ Channel Isoforms in Calyx Endings

We have found *Na_V_1.1–1.3, Na_V_1.5–1.6, Na_V_1.8*, and *Na_V_1.9* at different membrane domains of vestibular afferent calyx endings (Figure 15).

*Domain 1—*As before (Lysakowski et al. 2011), we found Na_V_1.5 at the CIF. Na_V_1.5, a typical cardiac isoform (Fozzard and Hanck 1996), is also expressed by developing vestibular ganglion cells (Liu et al. 2016) and immature calyces (Meredith and Rennie 2018). During excitatory stimulation, depolarization of the synaptic cleft hyperpolarizes the CIF (Domain 1) relative to other domains (see Figure 3C, Govindaraju et al. 2023). The Na_V_1.5 window current occurs at relatively negative voltages (−85 mV in cardiac muscle, −70 mV in neurons) and has a small‐amplitude peak (Moreau et al. 2013); this depolarizing current could help set membrane resting potential, augment EPSPs, and lead to spike initiation, as it does in olfactory sensory neurons (Frenz et al. 2014).

*Domain 2—C* afferent calyces are typically shorter than *D* calyces and either lack or have a truncated Domain 2. Na_V_1.9, found in Domain 2 of *D* calyces, has very slow kinetics and a large window current activating at relatively hyperpolarized voltages (Dib‐Hajj et al. 2015); Na_V_1.9 likely augments EPSPs and the rise to threshold rather than spike amplitude.

*Domain 3—*Na_V_1.3 generates large ramp currents during slow depolarizations and is thought to lower firing threshold and increase firing frequency (Cummins et al. 2001), thus Na_V_1.3 could hasten AP generation by graded potentials driven by excitatory transmission from hair cells and window currents from CIF Na_V_1.5. We previously predicted that increased calyx height enhances NQT and graded potentials in the calyx (Govindaraju et al. 2023). Na_V_1.3 expression in *C* calyces may be an adaptation to retain similar AP latencies as *D* units expressing Na_V_1.2, despite the shorter calyx height. Collectively, Domains 2 and 3 are typically 15–40 µm in length, similar to the length of an AIS in CNS.

*Domain 4—*In most *C* heminodes, only Na_V_1.1 is present; some also express Na_V_1.8. Na_V_1.1 and Na_V_1.6 colocalize at *D* heminodes. Such colocalization also occurs at heminodes of cochlear fibers innervating inner hair cells (K. X. Kim and Rutherford 2016) and in the AIS of many CNS neurons (Duflocq et al. 2008). The absence of Na_V_1.6 at *C* heminodes is somewhat surprising, given its ubiquitous presence at heminodes elsewhere. Na_V_1.1 reportedly carries less resurgent current than Na_V_1.6 (Patel et al. 2015). Na_V_β4 subunits are more prevalent at *D* afferent heminodes (Figure 11) and can enhance resurgent current through Na_V_1.6 (Barbosa et al. 2015) by increasing the window current and shifting the peak probability in a more hyperpolarizing direction (Zhao et al. 2011). Our identification of Na_V_1.6 in *D* heminodes (and absence in *C* heminodes) agrees with previous observations that much of the resurgent I_Na_ in PZ dimorphic fibers in gerbil crista slices is through Na_V_1.6 channels. This was demonstrated using 4,9‐ah‐TTX by Meredith and Rennie (2018, 2020), who found that this Na_V_1.6 subunit‐specific toxin only blocked ∼8% of Na_V_ current in crista CZ calyx‐only afferents in mature animals, indicating that mature CZ calyx‐only afferents contained something other than Na_V_1.6. Our results suggest that it could be Na_V_1.1 or Na_V_1.8, as we have found in the present study. Resurgent currents are associated with the ability to spike repetitively or in bursts (Lewis and Raman 2014). The presence of Na_V_1.6 and Na_V_β4 in *D* afferents could support their high firing rates (Goldberg 2000). Conversely, the preference for Na_V_1.1 in *C* afferents may reduce burst activity to preserve temporal fidelity.

*Domain Variable*—We found Na_V_1.8 at *C* heminodes, and at times throughout lightly stained calretinin‐positive *C* afferent terminals. Rarely, Na_V_1.8 intensely labeled what appeared to be a CIF in the peripheral zone, presumably belonging to a *D* afferent. Such labeling could be on type I hair cells, but due to the rarity of these Na_V_1.8‐labeled structures (8–9 out of hundreds of cells and mostly in the peripheral zone), we could not perform EM to resolve hair cell or calyx labeling. While not quantitative, PCR data (Wooltorton et al. 2007; Liu et al. 2016) suggest that levels of Na_V_1.4, Na_V_1.7, and Na_V_1.8 diminish by P21 in the vestibular epithelium and/or ganglion. Our lack of Na_V_1.4 and Na_V_1.7 immunostaining, as well as the variable staining of Na_V_1.8, may reflect this age‐related loss.

### 5.2 AnkG and AnkB Distributions Are Varied in Calyx Terminals

AnkG expression varied between *C* and *D* afferents. Notably, AnkG is relatively absent at the CIF in immunogold labeling, which is still a bit of a mystery; tenascin, contactin, and Na_V_β‐subunits could instead anchor the Na_V_1.5 channel (Lysakowski et al. 2011) as shown previously (Srinivasan et al. 1998). AnkG in the lateral membranes of supporting cells may contribute to their columnar nature—as in other epithelial cells that have been shown to remain flat otherwise (V. Bennett and Healy 2009).

*D* afferents were more extensively labeled for AnkB, including the COF (Domain 3), down to the hemi‐paranode and intraepithelial plexus; this is comparable to AnkB labeling of peripheral somatosensory fibers (Engelhardt et al. 2013). In vestibular fibers, AnkB labels the entire dendritic tree. By contrast, in Meissner's and Pacinian corpuscles, it labels the entire unmyelinated nerve inside the corpuscle, as well as the unmyelinated nerve portion of hair follicle receptors (Engelhardt et al. 2013).

AnkB has also been associated with Na^+^‐K^+^ ATPase in the heart (Mohler et al. 2005) and retina (Kizhatil et al. 2009). The α3 isoform of Na^+^‐K^+^ ATPase is present at high levels in vestibular sensory epithelium (ten Cate et al. 1994; Peters et al. 2001) and specifically localized on the outer surface of calyx endings of both *C* and *D* fibers (Schuth et al. 2014). It is possible that AnkB, in addition to its typical anchoring of K^+^ channels (Ogawa and Rasband 2008) at the hemi‐paranode in both *C* and *D* fibers, also anchors ATPase on the COF of *D* afferents.

### 5.3 HCN Channels Are Present on Both CIF and COF

HCN channels contribute to setting the resting membrane potential (He et al. 2014); current through these channels (I_h_) is present in type I hair cells (Rüsch et al. 1998; Horwitz et al. 2011) alongside I_KL_, a large outward rectifier current (Eatock and Songer 2011; Martin et al. 2024). I_h_ also occurs in calyx endings (Meredith et al. 2012, 2023; Songer and Eatock 2013; Horwitz et al. 2014; Contini et al. 2017). HCN1 is highly expressed in hair cells (Horwitz et al. 2014), whereas HCN2 is the dominant form in ganglion cells and calyx endings (Horwitz et al. 2014; Meredith et al. 2023). Whether HCN2 was situated on the CIF or COF was unclear in these earlier studies; we show it is present on both surfaces.

### 5.4 Most Heminodes Within the Vestibular Neuroepithelia Belong to *C* Fibers

That myelin could penetrate the basement membrane of the sensory neuroepithelium in mammals, was noted in early work by Wersäll and colleagues (Wersäll et al. 1954; Wersäll 1956; Wersäll and Bagger‐Sjöbäck 1974), and was established by (Ross et al. 1991), who called such axons “*M*” (myelinated) fibers, distinguishing them from “*M/U*” or “*U*” (unmyelinated) fibers, whose myelin ended at various levels below the basement membrane in the stroma. We found that most fibers with heminodes and myelin within the neuroepithelium were *C* afferents. Whether this is simply due to the reduced height of the epithelia in the striolar and central zones, or a functional adaptation, merits further investigation.

### 5.5 The Vestibular Calyx Terminal Is Akin to an AIS and its Heminode

Following excitatory transmission from hair cells, ongoing afferent depolarization is likely augmented by activation of Na_V_1.2 and Na_V_1.3 channels in Domain 3 of *C* and *D* units, respectively. This behavior enhances opening of Na_V_1.1 and Na_V_1.6 channels at the heminode. Na_V_1.6 has a lower firing threshold than Na_V_1.2 (Hu et al. 2009; Kole and Stuart 2012; Tian et al. 2014) and may be the first to open. Collectively, the different isoforms appear poised to initiate and shape the amplitude and timing of the AP (Figure 15); their distribution over tens of micrometers resembles that seen at the AIS of CNS neurons (Hu et al. 2009; Kole and Stuart 2012; Tian et al. 2014). For this reason, we propose that Domain 3 is akin to an AIS and that Domain 4, the heminode, is the spike initiation zone of vestibular *C* and *D* afferents.

## 6 Conclusion

The distribution of Na_V_1.1–1.3, 1.5, 1.6, 1.8, and 1.9 in vestibular calyx terminals suggests that the outer membrane and heminode act as the AIS and spike initiation zone, respectively. These isoforms may underlie three patterns of spiking (phasic, spontaneous, and oscillatory) and sodium currents (transient, persistent, and resurgent) observed in electrophysiological recordings of vestibular calyces (Meredith and Rennie 2020; Meredith et al. 2023) and characterized by TTX sensitivity in afferent calyx‐bearing fibers (Meredith and Rennie 2018) and vestibular ganglion cells (Liu et al. 2016). However, isoform‐specific current kinetics and conductance, which would permit detailed modeling, are not yet available for the microdomains we discuss here. Unraveling the functional significance of these isoforms for vestibular encoding through a combined approach using imaging, electrophysiology, and modeling should help us understand normal operation and design treatments for vestibular disorders based on ion channelopathies and further develop protocols for galvanic vestibular stimulation (Steinhardt and Fridman 2021) and vestibular implants.

## Author Contributions

**Anna Lysakowski**: conceptualization (equal), data curation, formal analysis (equal), funding acquisition, methodology, project administration, resources, supervision, validation, visualization, writing – original draft (equal), writing – review and editing (equal). **Aravind Chenrayan Govindaraju**: formal analysis (supporting), funding acquisition, visualization, writing – review and editing (supporting). **Steven D. Price**: data curation, investigation (lead), methodology, resources; **Sophie Gaboyard‐Niay**, investigation (supporting), methodology. **Irina Calin‐Jageman**: investigation (supporting), methodology. **Robstein L. Chidavaenzi**: investigation (supporting), methodology. **Ruth Anne Eatock**: conceptualization (supporting), funding acquisition, writing – review and editing (supporting). **Robert M. Raphael**: formal analysis (supporting), funding acquisition, writing – review and editing (supporting). **Jay M. Goldberg**: conceptualization (equal), formal analysis (equal), funding acquisition, resources, supervision, validation, writing – original draft (equal), writing – review and editing (equal).

## Funding

Financial support was provided by NIH R01 DC002058, NIH R01 DC012347, and R21‐DC022412.

## Conflicts of Interest

The authors declare no conflicts of interest.

## Significance Statement

Peripheral vestibular neurons form large chalice‐shaped nerve endings (calyces), which synapse with sensory hair cells that detect head motion. These neurons convert signals received from hair cells into action potentials that inform the brain. The distribution of ion channels in different regions of the calyx is an important factor contributing to spike initiation. We show that voltage‐gated sodium (Na_V_) channel isoforms, characteristic of central neuron axon initial segments (AISs) and spike initiation zones, are found in distinct regions of these nerve endings. We also note differences in Na_V_ isoforms between calyx terminals of neurons with additional bouton terminals (dimorphic) and without (calyx‐only) that likely contribute to different firing patterns in these terminals.

## Data Availability

Data available on request from the corresponding author.
